# Uncovering a possible role of reactive oxygen species in magnetogenetics

**DOI:** 10.1038/s41598-020-70067-1

**Published:** 2020-08-04

**Authors:** Matthew I. Brier, Jordan W. Mundell, Xiaofei Yu, Lichao Su, Alexander Holmann, Jessica Squeri, Baolin Zhang, Sarah A. Stanley, Jeffrey M. Friedman, Jonathan S. Dordick

**Affiliations:** 1 Department of Chemical and Biological Engineering, and Center for Biotechnology and Interdisciplinary Studies, Rensselaer Polytechnic Institute, Troy, NY 12180 USA; 2 Laboratory of Molecular Genetics, Rockefeller University, New York, NY 10065 USA; 3 State Key Laboratory Breeding Base of Nonferrous Metals and Specific Materials Processing, College of Material Science and Engineering, Guilin University of Technology, Jian Gan Road 12, Guilin, 541004 China; 4 Diabetes, Obesity and Metabolism Institute, Icahn School of Medicine At Mount Sinai, New York, NY 10029 USA; 5 Howard Hughes Medical Institute, New York, NY 10065 USA; 6 Departments of Biomedical Engineering and Biological Sciences, Rensselaer Polytechnic Institute, Troy, NY 12180 USA; 7 Present Address: State Key Laboratory of Genetic Engineering, School of Life Sciences, Fudan University, Shanghai, 200438 China

**Keywords:** Biotechnology, Ion channels, Synthetic biology

## Abstract

Recent reports have shown that intracellular, (super)paramagnetic ferritin nanoparticles can gate TRPV1, a non-selective cation channel, in a magnetic field. Here, we report the effects of differing field strength and frequency as well as chemical inhibitors on channel gating using a Ca^2+^-sensitive promoter to express a secreted embryonic alkaline phosphatase (SEAP) reporter. Exposure of TRPV1-ferritin-expressing HEK-293T cells at 30 °C to an alternating magnetic field of 501 kHz and 27.1 mT significantly increased SEAP secretion by ~ 82% relative to control cells, with lesser effects at other field strengths and frequencies. Between 30–32 °C, SEAP production was strongly potentiated 3.3-fold by the addition of the TRPV1 agonist capsaicin. This potentiation was eliminated by the competitive antagonist AMG-21629, the NADPH oxidase assembly inhibitor apocynin, and the reactive oxygen species (ROS) scavenger *N*-acetylcysteine, suggesting that ROS contributes to magnetogenetic TRPV1 activation. These results provide a rational basis to address the heretofore unknown mechanism of magnetogenetics.

## Introduction

New approaches have been advanced for controlling signal transduction^1^, cell activity, and protein expression^2^ with temporal precision, contributing to advances in on-demand biomanufacturing of protein biologics^3^, developing new tools for drug discovery^4^, in vitro expansion and differentiation of stem cells for regenerative medicine^5^, and regulating the activity of neurons and other cell types in vivo^5^. One example is optogenetics, which uses precise wavelengths of light to stimulate light sensitive channels^6,7^. Alternatively, gold nanorods (AuNRs) can be actuated by near-infrared (NIR) wavelengths to confer light-sensitivity to light-insensitive, heat-sensitive targets. Specifically, antibody-coated AuNRs targeted to transient receptor potential (TRP) vanilloid 1 (TRPV1) cation channels and integrins generated plasmonic heating when stimulated with select wavelengths of NIR light and gated Ca^2+^ flux into cells to control cellular functions^8^. In each optical technique, the low penetration depth of visible and NIR light into cellular systems limits application either in vitro or in vivo^9^. Another example is chemogenetics, in which drug ligands can gate mutated ion channels^10^ or G-protein coupled receptors^11^. Chemogenetics does not require an implant but is limited by a slow onset of action that is dictated by the pharmacokinetics of the drug actuator in vivo and the addition of a chemical inducer to in vitro cell-based reactors^12^. Still other approaches for activating signal transduction have been developed including the use of size-controlled microbubbles targeted to Piezo1 ion channels to gate Ca^2+^ flux by ultrasound stimulation^13^ and magnetic activation of engineered ion channels^14–18^.

The use of magnetic fields to gate TRPV1 and related ion channels has been shown in multiple studies. Pralle and co-workers used megahertz radio frequency (RF) alternating magnetic fields (AMFs) to gate TRPV1 when external magnetic nanoparticles were localized to the cell surface^19^. Similarly, Stanley et al. demonstrated that TRPV1 could be activated remotely by external superparamagnetic iron oxide nanoparticles (SPIONs) tethered to the first extracellular loop of TRPV1 in a 465 kHz, 29–32 mT RF-AMF^14^. This remote activation resulted in Ca^2+^ flux into cells and expression of proinsulin in vitro and decreased blood glucose levels in vivo using a synthetic Ca^2+^-dependent promoter to drive transgene expression. In analogous studies, magnetic fields have been applied to gate TRP channels tethered to endogenously expressed ferritin nanoparticles. For example, expression of genetically-encoded, intracellularly-expressed ferritin nanoparticles tethered to TRPV1 were shown to elicit quantitatively similar magnetogenetic effects as SPIONs when exposed to 465 kHz, 29–32 mT RF-AMFs both in vitro and in vivo^15,16^. In separate studies, Mosabbir and Truong demonstrated that a pulsed 10 mT magnetic field could actuate ferritin-tagged TRPV1, gate calcium flux, and stimulate cell migration^17^. Likewise, Güler and colleagues used a ferritin fused to the C-terminus of TRPV4 to gate Ca^2+^ flux and elicit biologic effects in vitro and in vivo^18^.

While these results show that ferritin-tethered ion channels can be gated by magnetic fields, the mechanism remains unclear. This uncertainty arises from a lack of understanding of how the magnetic ferritin nanoparticle translates magnetic fields into stimuli that activate target TRP channels, which are sensitive to an array of orthogonal stimuli. For example, the multimodal non-selective cation channel TRPV1 is known to be gated by temperature (> 42 °C)^20,21^, pH (< 5.2)^22^, voltage (positive potentials > 100 mV)^21^, mechanical force^23^, and chemical agonists (e.g., capsaicin and resiniferatoxin)^20^. As such, this lack of a mechanistic understanding limits rational application of magnetogenetics. To this end, several possible physical mechanisms have been proposed, including nanoparticle-driven heating, but have also been questioned^24^.

Here, we use secreted embryonic alkaline phosphatase (SEAP) under the control of a Ca^2+^-dependent promoter as a reporter to explore AMF-induced activation of ferritin-tethered TRPV1 in vitro. Significant increase in SEAP secreted into the medium was observed in response to specific AMF field strength and frequency parameters. Surprisingly, significant potentiation of SEAP production was observed in the presence of both AMF and the TRPV1 agonist capsaicin, and diminution of SEAP production was observed in the presence of inhibitors of reactive oxygen species (ROS) generation. Interestingly, prior studies have shown that free iron can lead to ROS, which can gate TRP channels^25–27^. In aggregate these results establish the importance of AMF strength and frequency on TRPV1 activation and suggest involvement of a chemical mechanism involving ROS generation in a magnetic field to control biological systems.

## Results

### Design of TRPV1-ferritin/SEAP reporter system

**Figure 1:**
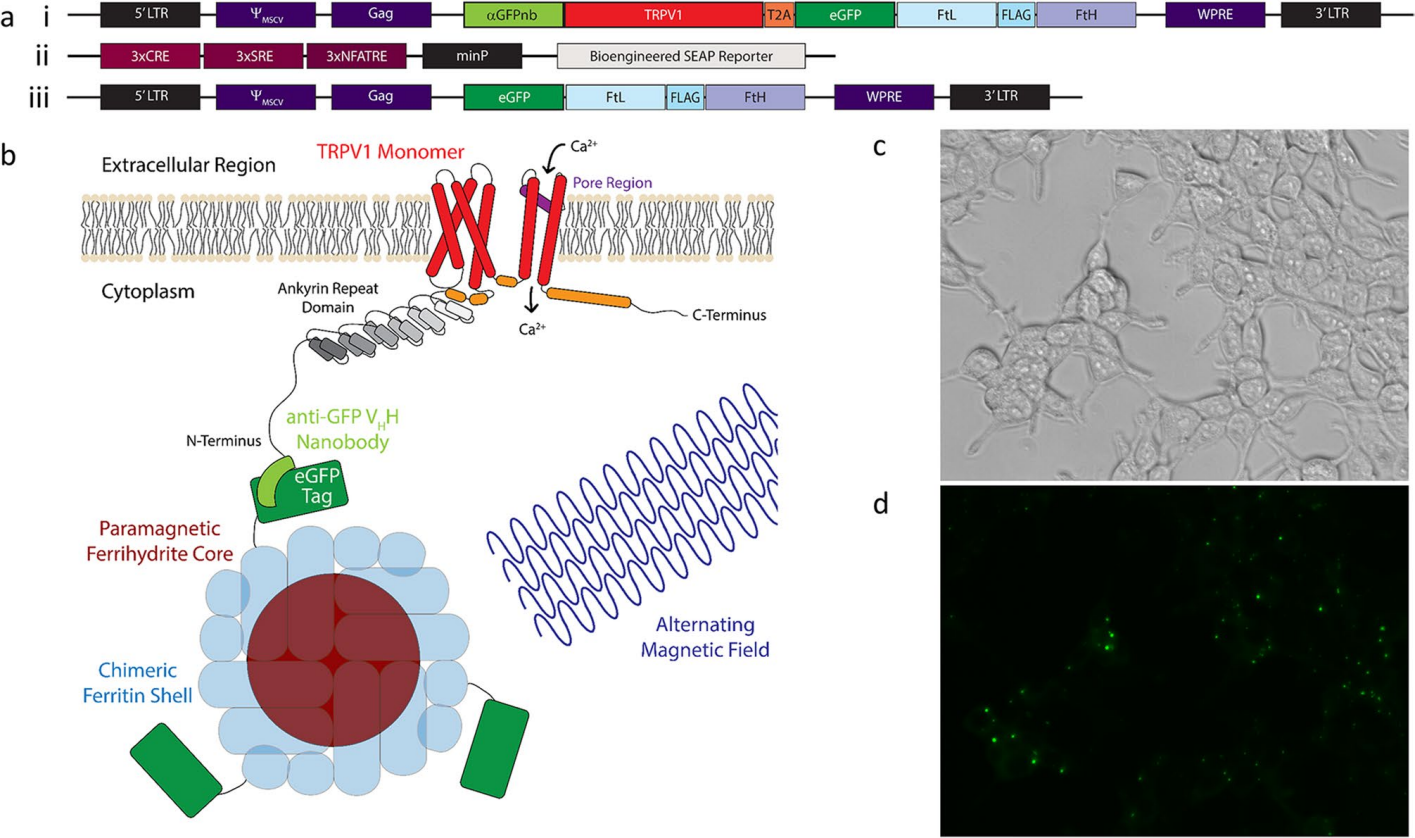
Magnetogenetic platform expressed in HEK-293T cells. (**a**, i–iii) Constructs used to express the magnetogenetic platform in HEK-293T cells. (**a**, i) Construct used to stably integrate the αGFPnb-TRPV1-T2A-eGFP-FtD platform and create a stable cell line for magnetogenetic stimulation studies. (**a**, ii) Construct used to transiently transfect the Ca^2+^-dependent SEAP reporter. (**a**, iii) Construct used to stably integrate the eGFP-FtD portion of platform and create a stable cell line for ferritin immunoprecipitation studies. (**b**) Cartoon schematic of a monomer of the αGFPnb-TRPV1 fusion conjugated to eGFP-tagged chimeric ferritin and activated through magnetic field stimulation to induce gating of calcium. (**c**) Brightfield and (**d**) fluorescent images of stably expressing nbV1/FtD cells showing expression and localization of the αGFPnb-TRPV1-T2A-eGFP-FtD construct (**a**, i) to cell membranes.

A rat TRPV1 cation channel with an N-terminally fused camelidae anti-green fluorescent protein (GFP) nanobody (αGFPnb)^15^ was stably co-expressed in HEK-293T cells together with an enhanced GFP (eGFP)-tagged ferritin light chain (FtL)-FLAG-tag-heavy chain (FtH) dimer (referred hereafter as eGFP-FtD)^28^ (Fig. 1a (i)). Previous studies have shown that ferritin dimers expressed from this construct are incorporated into chimeric, eGFP-tagged ferritin nanoparticles through integration of transfected ferritin subunits with endogenous FtL and FtH chains into complete 24-subunit ferritin^15,16,28^. A T2A cleavage site was included to ensure relatively equal expression of αGFPnb-TRPV1 and eGFP-FtD. The αGFPnb-TRPV1-T2A-eGFP-FtD construct (or nbV1/FtD) was then incorporated into a murine stem cell virus (MSCV) vector for stable integration and constitutive expression in a HEK-293T stable cell line (referred to as HEK-nbV1/FtD), which did not alter cell proliferation. TRPV1 is a homotetramer and co-expression of eGFP-ferritin with αGFPnb-TRPV1 results in a membrane complex consisting of TRPV1 tethered to ferritin, with the potential for one to four ferritins per tetrameric TRPV1^15^ (Fig. 1b). Brightfield microscopy of HEK-nbV1/FtD cells showed normal morphology (Fig. 1c and Supplementary Fig. 1a) and fluorescence microscopy confirmed expression of functional αGFPnb-TRPV1 tetramers (Supplementary Fig. 2) with eGFP-FtD integrated ferritin in transiently transfected HEK-293T (Supplementary Fig. 1b) and HEK-nbV1/FtD cell membranes (Fig. 1d).

We used a Ca^2+^-sensitive reporter gene comprised of a synthetic Ca^2+^-dependent promoter containing three triplicate response elements for serum (SRE), cyclic adenosine monophosphate (cAMP, CRE), and nuclear factor of activated T-cells (NFATRE), followed by a minimal promoter (minP) upstream of a SEAP cDNA (Fig. 1a (ii)). The Ca^2+^-dependent SEAP reporter (CaR-SEAP) was previously validated using an insulin cDNA as reporter^14,15^, and in the current study SEAP allowed direct and rapid quantification of gene expression via a simple colorimetric assay.

### Structural properties of expressed ferritin

**Figure 2:**
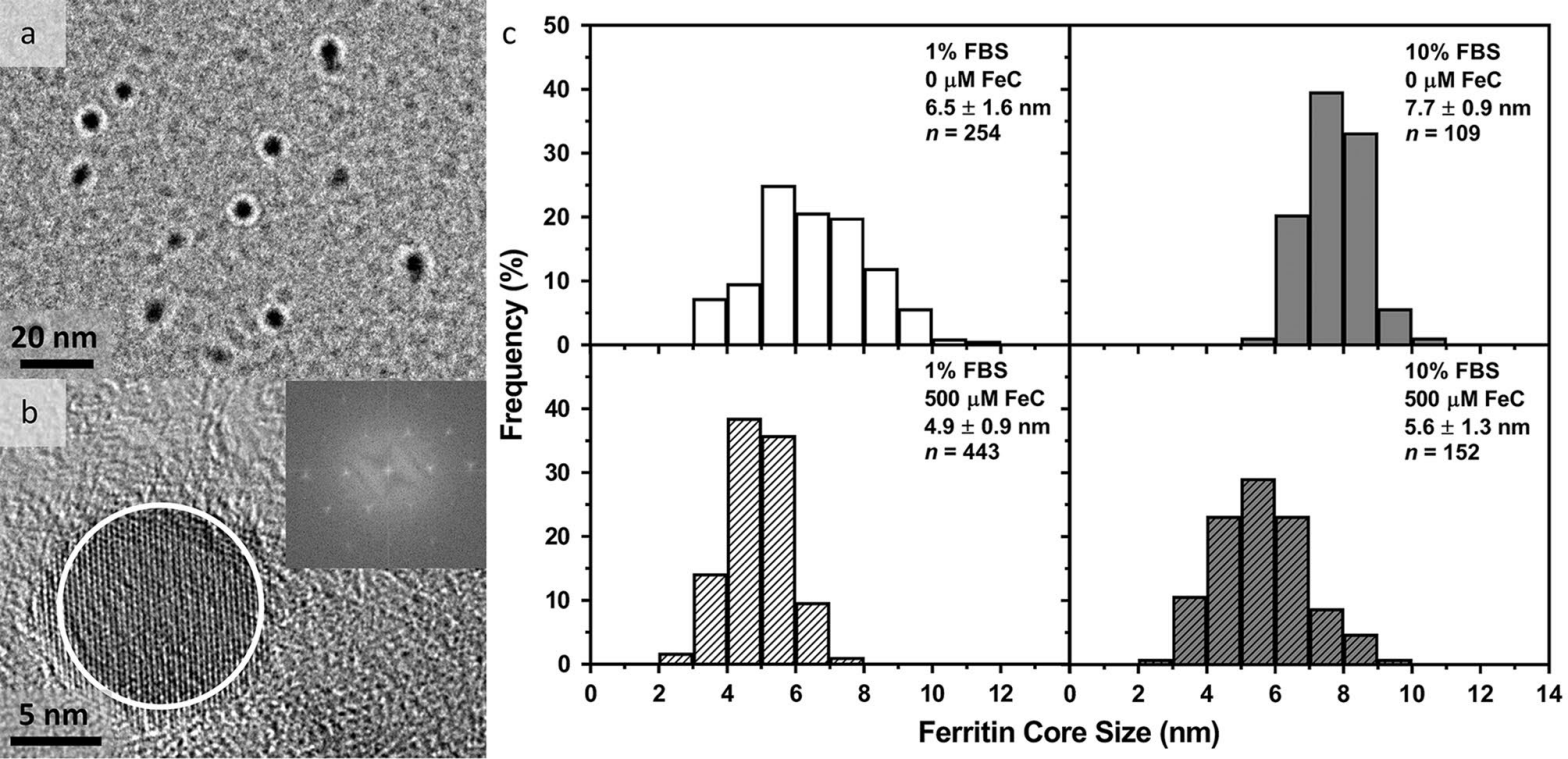
Characterization of ferritin produced in HEK-293T cells. TEM images and nanoparticle core size distributions from FLAG-tag immunoprecipitation. (**a**) TEM image of phosphotungstic acid stained ferritin nanoparticles purified from HEK-FtD cells grown in media supplemented with 10% FBS, 0 mg⋅mL^−1^ HTF, and 500 μM FeC. (**b**) HRTEM image of an unstained ferritin nanoparticle collected from HEK-FtD cells grown in media supplemented with 1% FBS, 0 mg∙mL^−1^ HTF, and 0 μM FeC. HRTEM shows a clear ED pattern of a highly crystalline lattice (inset using region circled in white) when analyzed using the ImageJ FIJI FFT function. (**c**) Core size distributions from unstained TEM images of ferritin nanoparticles collected from HEK-FtD cells grown under four growth medium conditions. TEM images were analyzed using the ImageJ FIJI nanoparticle size function, in which a minimum of 100 nanoparticles were measured, and distributions were normalized by their respective total nanoparticle counts. Statistical significance for differences in ferritin core size distributions shown in (**c**) was calculated using two-way ANOVA with Tukey’s multiple comparisons test, which found a significant two-way interaction between FBS and FeC concentration in growth media on core size, *F*(1,1,051) = 8.896, *p* = 0.0029.

Ferritin essentially serves as a “transceiver” of the magnetic field. Chemical loading of ferritin with iron ex vivo is known to impact core size distribution, crystallinity, and magnetic properties^29^. To this end, the eGFP-FtD construct (Fig. 1a (iii)) was cloned into an MSCV vector by itself for constitutive expression in HEK-293T cells (referred to as HEK-FtD). The HEK-FtD cells were grown in media supplemented with fetal bovine serum (FBS, 1 or 10%), holo-transferrin (HTF, 0 or 2 mg∙mL^−1^), and ferric citrate (FeC, 0 or 500 μM). eGFP-tagged ferritin was precipitated using an anti-FLAG antibody and the size and crystal structure of the immunopurified ferritin were examined using transmission electron microscopy (TEM). TEM images of the purified ferritin nanoparticles stained with the heavy metal reagent phosphotungstic acid revealed clear ferritin cores (black circular regions) surrounded by a protein cage (white annuli surrounding the cores) (Fig. 2a). Consistent with prior studies^30^, these images showed an average ferritin cage thickness of 2.4 ± 0.4 nm.

HRTEM of unstained ferritin showed a nanoparticle core consisting of an apparent single-crystal structure (Fig. 2b) as revealed in an electron diffraction (ED) pattern extracted using fast Fourier transform (FFT) image analysis (Fig. 2b, inset). This highly crystalline structure was observed in ferritin nanoparticles from cells with the lowest expected iron content (cultured with no added iron or HTF: 1% FBS, 0 mg∙mL^−1^ HTF, and 0 μM FeC) and the highest expected iron content (cultured with added iron and transporters: 10% FBS, 2 mg∙mL^−1^ HTF, and 500 μM FeC). The presence of higher serum and FeC concentrations in the absence of HTF impacted ferritin core size distributions (Fig. 2c). In the absence of both HTF and FeC, increasing the serum concentration of the cultured cells from 1 to 10% resulted in an 18% increase in core size distribution. In the absence of HTF but in the presence of 500 μM FeC, the increase in serum from 1 to 10% resulted in a 14% increase in core size (Fig. 2c). In contrast to the effect of serum on increased core size, higher bioavailable iron concentrations decreased core size. Specifically, in 1% serum the addition of 500 μM FeC resulted in a 25% decrease in core size distribution, while in 10% serum the presence of 500 μM FeC resulted in a 27% decrease in core size distribution. Thus, increased FBS concentration resulted in increased core size while increased FeC concentration yielded decreased core sizes. As a result, we used a combination of 10% FBS, 2 mg∙mL^−1^ HTF, and 500 μM FeC during the preparation of HEK-nbV1/FtD cells to drive iron loading and core formation of ferritin for subsequent studies. These conditions enabled a more compact ferritin iron core with higher iron density while using a higher serum concentration to aid in cell metabolism.

### Effect of alternating magnetic field strength and frequency on SEAP production

**Figure 3:**
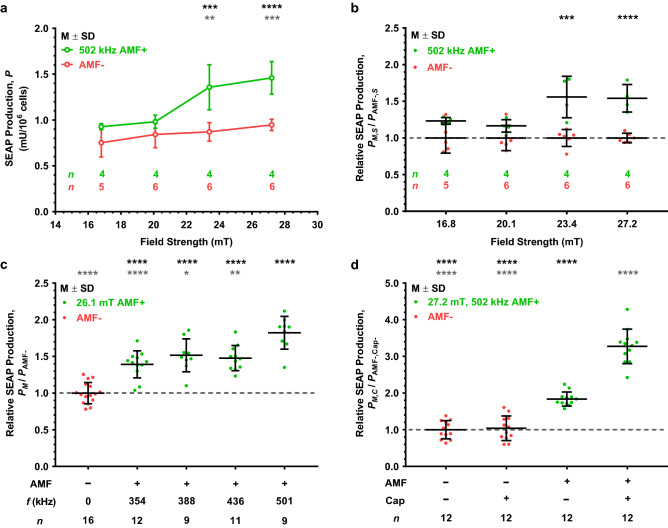
Magnetogenetic platform response to AMF stimulation as a function of field strength, frequency, or presence of capsaicin. (**a**) SEAP production as a function of average modeled AMF strength (black circles) compared to corresponding no treatment temperature-matched controls (gray circles). AMF stimulation performed at 502 kHz for 1 h. Temperature-matched controls varied by stimulation strength due to increasing power requirement for higher AMF strengths. Black and gray asterisks denote statistical significance above ~ 20.1 mT average stimulation condition and respective AMF− controls, respectively. (**b**) Relative SEAP production for each AMF strength with respect to its corresponding no treatment control (denoted as a dashed line) plotted in (**a**). Significance noted for two highest stimulation conditions over the respective AMF− control. (**c**) Relative SEAP production of each AMF stimulation frequency with respect to the AMF− control at 30 °C. All frequencies tested at an average modeled AMF strength of 26.1 mT for 2 h. Black and gray asterisks denote significance with respect to the AMF− control and the 501 kHz AMF stimulation conditions, respectively. (**d**) Relative SEAP production upon capsaicin (Cap) potentiation. AMF stimulation performed at 502 kHz and ~ 27.2 mT for 2 h. Both AMF+ groups were significantly more responsive than their AMF− counterparts, and the AMF+/Cap+ potentiation group (significant comparisons denoted with black asterisks) was significantly more responsive than the AMF+/Cap− group (denoted with gray asterisks). Values displayed as mean ± standard deviation (M ± SD). Statistical significance for (**a,b,d**) using two-way ANOVA with Tukey’s multiple comparisons test and (**c**) using one-way ANOVA with Šídák’s multiple comparisons test. (**a,b**) Significant two-way interaction between AMF treatment and field strength, *F*(3,31) = 5.082, *p* = 0.0056. (**c**) Significant effect due to AMF frequency, *F*(5,66) = 24.737, *p* < 0.0001. (**d**) Significant two-way interaction between treatment with AMF and capsaicin, *F*(1,44) = 54.13, *p* < 0.0001. Significance level denoted using asterisks (*): * *p* < 0.05, ** *p* < 0.01, *** *p* < 0.001, and **** *p* < 0.0001.

A custom RF induction system with a 2-turn coil was used to vary both frequency and field strength, from approximately 350 to 500 kHz and approximately 5 to 34 mT, respectively (see Supplementary Information and Supplementary Fig. 3–5 for details). At a frequency of 502 kHz, SEAP production was increased relative to the basal level as a function of field strength in a statistically significant, step-wise fashion (Fig. 3a,b). We observed a small, but not statistically significant, increase in SEAP production in control samples not exposed to the AMF (AMF−). This small increase is likely due to a small temperature increase from approximately 26 to 32 °C caused by the increased power applied to the coil and measured during temperature equilibration of the coil between treatments at each field strength. There was also a small, yet significant effect of frequency in the range from ~ 354 to ~ 501 kHz at an average field strength of ~ 26.1 mT (Fig. 3c). All four frequencies tested in the 354–501 kHz range showed a significant increase of SEAP production over the basal AMF-condition at 30 °C (Fig. 3c, black asterisks) with the highest level of SEAP production relative to the AMF-condition at 501 kHz (Fig. 3c). Finally, a field strength of ~ 17.1 mT (not shown) was also tested using these same frequencies and only minimal amounts of SEAP were secreted relative to basal conditions, reconfirming the requirement of a field strength at or above ~ 23.4 mT for ferritin-mediated TRPV1 activation in a magnetic field.

Capsaicin is an extensively studied TRPV1 agonist and has previously been shown to gate TRPV1 in heterologous cells in vitro using electrophysiology and calcium imaging^20,21^. Capsaicin sensitizes TRPV1 by reducing its temperature-dependent activation “threshold”, as observed by micromolar doses gating channels at room temperature (well below that of its normal activation at 42–43 °C) in fluorescent calcium imaging (Supplementary Fig. 2) and electrophysiology studies^20,21^. Thus, we tested for a possible synergistic effect of capsaicin and a magnetic field at a coil temperature of ~ 30–32 °C by adding 1.0 μM capsaicin (Cap+) to cultured cells while exposing them to 502 kHz AMF stimulation at ~ 27.2 mT (AMF+) (Fig. 3d). Even though 1.0 μM capsaicin sensitizes gating of TRPV1 and is readily observed at temperatures close to 37 °C (Supplementary Fig. 6a,c,e), this same dose of capsaicin had no discernible effect on cells at 30–32 °C in the absence of AMF (AMF−/Cap+) relative to the basal response condition (AMF−/Cap−). In contrast, at ~ 30–32 °C the level of SEAP generated by AMF stimulation (AMF+/Cap−) was significantly greater than under basal conditions or after treatment with capsaicin alone. Interestingly, the combination of AMF and capsaicin (AMF+/Cap+) resulted in a dramatic potentiation of TRPV1 gating with a ~ 230% increase over the basal condition, ~ 210% increase over capsaicin alone, and ~ 78% increase over AMF stimulation alone. This observation is consistent with previous studies of TRPV1 regarding potentiation of channel opening by combinations of heat, voltage, pH, and chemical ligands including capsaicin^20,21,31^.

### Exploring a chemical mechanism of magnetogenetics

AMFs are capable of inducing ROS formation at the surface of iron oxide nanoparticles^32^ as well as accelerating the release of iron ions from iron sequestering proteins such as ferritin^33^, which could result in localized ROS generation including H_2_O_2_ around the ferritin nanoparticles. H_2_O_2_, in particular, has been reported to oxidize specific cysteine residues in the C-terminal domain of TRPV1 and in turn potentiate capsaicin activation^25^. Therefore, we considered that AMFs might increase Ca^2+^ influx due to TRPV1 gating by ferritin-generated ROS. We hypothesize that this could lead to further generation of ROS by activating protein kinase C (PKC), specifically isoforms α and βII, which in turn would initiate the assembly of NADPH oxidase (NOx) isoform 1 in HEK-293T cells^34^. Activated NOx would generate H_2_O_2_, leading to a cascade of downstream calcium signaling events.

This potential cascade of events was first probed by assessing the effect of chemical modulators on SEAP production in HEK-nbV1/FtD exposed to a range of capsaicin doses. Dose dependent TRPV1-mediated SEAP production was observed with respect to both capsaicin and inhibitor concentration for all three inhibitors (Supplementary Fig. 6). In the presence of AMG-21629, a potent competitive inhibitor of TRPV1 activation by capsaicin^35,36^, dose-dependent inhibition of SEAP production was observed at 0.50 and 1.0 μM capsaicin resulting in IC_50_ values of 1.5 ± 0.2 and 5.2 ± 0.4 nM (M ± SEM), respectively (Supplementary Fig. 6a,b). Dose-dependent inhibition of SEAP production was also observed with apocynin, a molecule that upon intracellular oxidation inhibits NOx assembly^37,38^, with IC_50_ values of 88 ± 15 and 140 ± 40 μM for 0.50 and 1.0 μM capsaicin, respectively (Supplementary Fig. 6c,d). Finally, dose-dependent inhibition of SEAP production was observed in the presence of *N*-acetylcysteine (NAC), an indiscriminate ROS scavenger^39^, with calculated IC_50_ values of 5.2 ± 1.8 and 4.3 ± 1.3 mM for 0.50 and 1.0 μM capsaicin, respectively (Supplementary Fig. 6e,f). Based on these results, concentrations of 100 nM AMG-21629, 100 μM apocynin, and 5.0 mM NAC were used to further study the role of ROS in the AMF-mediated induction of SEAP production.

**Figure 4:**
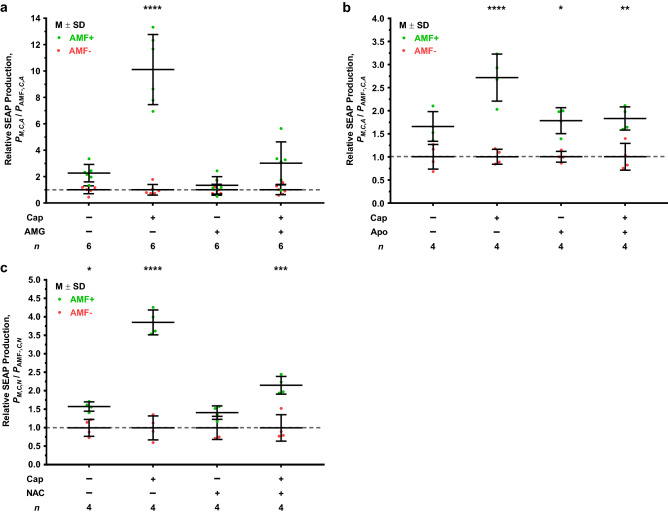
Inhibition of magnetogenetic platform response to AMF and AMF/Cap potentiated stimulation. (**a**) Relative SEAP production for AMF+ (~ 27.1 mT, 502 kHz AMF) over AMF− (32.0 °C) for each capsaicin/AMG-21629 combination group during a 2 h treatment period. Capsaicin (Cap+: 1.0 μM) and AMG-21629 (AMG+: 100 nM) were used to potentiate and inhibit, respectively, AMF stimulation. (**b**) Relative SEAP production for AMF+ (~ 27.4 mT, 502 kHz AMF) over AMF− (32.0 °C) for each capsaicin/apocynin combination group during a 2 h treatment period. Capsaicin (Cap+: 1.0 μM) and apocynin (Apo+: 100 μM) were used to potentiate and inhibit, respectively, AMF stimulation. (**c**) Relative SEAP production for AMF+ (~ 27.2 mT, 502 kHz AMF) over AMF− (32.0 °C) for each capsaicin/*N*-acetylcysteine combination group during a 2 h treatment period. Capsaicin (Cap+: 1.0 μM) and *N*-acetylcysteine (NAC+: 5.0 mM) were used to potentiate and inhibit, respectively, AMF stimulation. Dashed line at value of 1 denotes the mean of the AMF− condition for each capsaicin/inhibitor combination that the AMF+ treatment is relative to after normalization. Values displayed as mean ± standard deviation (M ± SD). Statistical significance for (**a–c**) was calculated using a three-way ANOVA with Tukey’s multiple comparisons test. (**a**) Significant three-way interaction between treatment with AMF, capsaicin, and AMG-21629, *F*(1,40) = 22.43, *p* < 0.0001. (**b**) Significant three-way interaction between treatment with AMF, capsaicin, and apocynin, *F*(1,24) = 4.67, *p* = 0.0409. (**c**) Significant three-way interaction between treatment with AMF, capsaicin, and *N*-acetylcysteine combination, *F*(1,24) = 6.475, *p* = 0.0178. Statistical significance is denoted for the AMF+ treatment of each capsaicin/inhibitor combination to its corresponding AMF− treatment group before normalization. Significance level denoted using asterisks (*): * *p* < 0.05, ** *p* < 0.01, *** *p* < 0.001, and **** *p* < 0.0001.

The effect of each of the aforementioned chemical modulators on SEAP production in the presence (+) and absence (−) of AMF and/or capsaicin are shown in Fig. 4 as the ratio of SEAP production for AMF+ relative to AMF− (AMF+/AMF−) for each capsaicin/inhibitor combination (Cap/Inhib). At 32 °C in the absence of AMF (AMF−), the addition of capsaicin alone without an inhibitor (Cap+/Inhib−) did not lead to a significant response over the basal condition (Cap−/Inhib−) (Supplementary Fig. 7a–c). Moreover, neither the presence of each of the three inhibitors without capsaicin (Cap−/Inhib+) nor the combination of capsaicin with each of the three inhibitors individually showed an effect over the basal condition. HEK-293T cells transiently transfected with the nbV1/FtD construct also showed AMF-induced SEAP production in the absence of capsaicin or inhibitor (Supplementary Fig. 7b,c; Cap−/Inhib−) and the response over baseline was similar to that of the HEK-nbV1/FtD stable cell line previously shown in Fig. 3d (Fig. 4b,c; AMF+/AMF− ≈ 2). Furthermore, the AMF+/AMF− ratios for the combined AMF-capsaicin-induced SEAP production in the absence of inhibitor for each of the different inhibitor tests (Fig. 4a–c; Cap+/Inhib−) were similar to the observed 2.1-fold increase for AMF+/Cap+ over AMF−/Cap+ (Fig. 3d), further demonstrating qualitative and quantitative reproducibility of the statistically significant potentiation of AMF-induced TRPV1 activation by capsaicin.

Consistent with the known ability of AMG-21629 to block capsaicin-induced gating of TRPV1, the ability of capsaicin to potentiate the effect of AMF was nearly completely inhibited in the presence of 100 nM AMG-21629 (Fig. 4a); the AMF+/AMF− ratio for capsaicin potentiation (Cap+/AMG−) dropped significantly from 10 ± 3 (M ± SD) to 3.0 ± 1.6 when inhibited with AMG-21629 (Cap+/AMG+). In the absence of capsaicin, 100 nM AMG-21629 (Cap−/AMG+) had no significant effect on SEAP production after exposure to AMF relative to baseline (Fig. 4a). While not statistically significant, there is a noticeable decrease in the AMF+/AMF− ratio from 2.3 ± 0.7 for Cap−/AMG− to 1.3 ± 0.7 for Cap−/AMG+, potentially indicating that binding of AMG-21629 to TRPV1 may lead to an inhibitory structural change that raises the threshold for AMF-induced activation^40,41^.

We next evaluated the effect of ROS modulators on the ability of AMF together with 1.0 μM capsaicin to induce SEAP production. Similar to AMG-21629, we observed an inhibitory effect with the addition of 100 μM apocynin; the ratio of SEAP production for AMF+/AMF− in the presence of 1.0 μM capsaicin (Cap+/Apo−) dropped from 2.7 ± 0.5 to 1.8 ± 0.3 when apocynin was added (Cap+/Apo+) (Fig. 4b). However, in the absence of capsaicin the addition of apocynin (Fig. 4B; Cap−/Apo+) had no effect on the AMF+/AMF− ratio and we did not observe the reduction in this ratio seen with Cap−/AMG+ (Fig. 4a). Together these results suggest that inhibiting ROS generation by NOx using apocynin also inhibits SEAP production, specifically in the presence of capsaicin, and that activation of NOx is downstream of the AMF-initiated gating interaction and likely requires greater calcium influx that only occurs when capsaicin is added (see Supplementary Fig. 8 and associated discussion).

The AMF+/AMF− ratio in the presence of 5.0 mM NAC and 1.0 μM capsaicin (Cap+/NAC+) dropped from 3.8 ± 0.3 (Cap+/NAC−) to 2.1 ± 0.2 when inhibited with NAC (Fig. 4c). Unlike the Cap−/Apo+ combination (Fig. 4b), addition of NAC without capsaicin (Fig. 4c; Cap−/NAC+) generated a small decrease in AMF+/AMF− ratio similar to Cap−/AMG+ (Fig. 4a) such that the effect of AMF on SEAP production was not significantly different from baseline. This result indicates that ROS scavenging is capable of blocking SEAP production during exposure of cells expressing nbV1/FtD to AMF and also blocks the potentiation with capsaicin. However, this NAC concentration (roughly the IC_50_ value from Supplementary Fig. 6f) was neither sufficient to achieve significant reduction in the AMF response in the absence of capsaicin nor did it completely quench the potentiated AMF-capsaicin response.

## Discussion

Magnetogenetic gating of ion channels employs a platform in which a (super)paramagnetic external or endogenously expressed internal nanoparticle is placed in proximity to a diamagnetic cell membrane receptor. In this configuration, the nanoparticle essentially acts as a transceiver, receiving an electromagnetic stimulus and transmitting it to the receptor (ion channel), which in turn can modulate cellular function. This gives magnetogenetic platforms spatiotemporal control analogous to other approaches including optogenetics and mechanogenetics. While several groups have reproduced the key observation that a sufficiently strong magnetic field can gate TRPV1^15–17^ and TRPV4^18,42^, some theoretical calculations have questioned whether ferritin’s magnetic properties are sufficient to actuate the channels by either localized heating or mechanical force^24^, as has been proposed. Additionally, follow-up studies of the TRPV4-ferritin platform by Wang et al*.*^43^, Xu et al.^44^, and Kole et al.^45^, all using electrophysiological readouts, have been unsuccessful in recapitulating the results by Wheeler et al.^18^, leading others to suggest alternative physical mechanisms that may account for the growing body of empiric data^42,46,47^. However, none of these studies proposed a possible chemical mechanism, a possibility that was tested here using chemical inhibitors or quenchers of reactive oxygen species generation.

Herein, we used expression of a Ca^2+^-dependent reporter gene to show that ~ 350–500 kHz AMFs gate TRPV1 that is tethered to genetically-encoded eGFP-complexed ferritin at the channel’s N-terminus by an anti-GFP nanobody at temperatures as low as 30 °C. At this temperature, in the absence of AMF stimulation, minimal reporter expression, and by extension TRPV1 gating, is observed. This finding confirms earlier reports that 465–470 kHz AMF enables gating of ferritin-tethered TRPV1^15,16^. Intriguingly, we also found that, similar to its ability to potentiate gating in response to a subthreshold temperature, the TRPV1 agonist capsaicin strongly potentiates TRPV1 gating by AMF, even under conditions where capsaicin alone has no effect. The mechanism of TRPV1 activation by AMF and potentiation in the presence of 1.0 μM capsaicin may have multiple contributors, both physical and chemical. With respect to the latter, capsaicin binds to TRPV1 inducing a conformational change^40,41^, which would reduce the energy needed to open the channel during AMF treatment and result in the observed enhancement in SEAP expression. Similarly, AMF treatment could sensitize the channel thereby reducing the temperature threshold for TRPV1 gating. In this case, ferritin-mediated AMF exposure would not gate TRPV1 directly, but could instead sensitize it through an orthogonal conformational change in the channel, which upon addition of capsaicin could further lower the temperature threshold to the 30–32 °C range where the channel would be predominantly in the open configuration. It is noteworthy that AMG-21629, a competitive inhibitor to capsaicin binding and TRPV1 activation, nearly eliminated reporter expression by AMF exposure (Fig. 4a). As AMG-21629 binds to the same pocket in TRPV1 as capsaicin, the reduction in response raises the possibility that the antagonist restricts a necessary conformational change, thus preventing AMF from either activating or sensitizing TRPV1 in a manner opposite to that in which capsaicin potentiates the interaction.

Addition of ROS inhibitors (either to ROS formation or by quenching) indicates involvement of ROS in TRPV1 gating by the combination of AMF and capsaicin. NADPH oxidase is a key component of a robust ROS generation system in mammalian cells and these data indicate that it plays an important role in TRPV1 channel gating. Inhibition of NOx by apocynin strongly supports a ROS-driven mechanism of TRPV1 activation in response to an AMF plus capsaicin. Indeed, superoxide radicals produced by NOx isoform 1 catalysis in HEK-293T cells would rapidly dismute to H_2_O_2_, which gates TRPV1^25^. This is further supported by the quenching studies with NAC, which inhibits combined AMF-capsaicin TRPV1 activation as assayed by SEAP expression.

**Figure 5:**
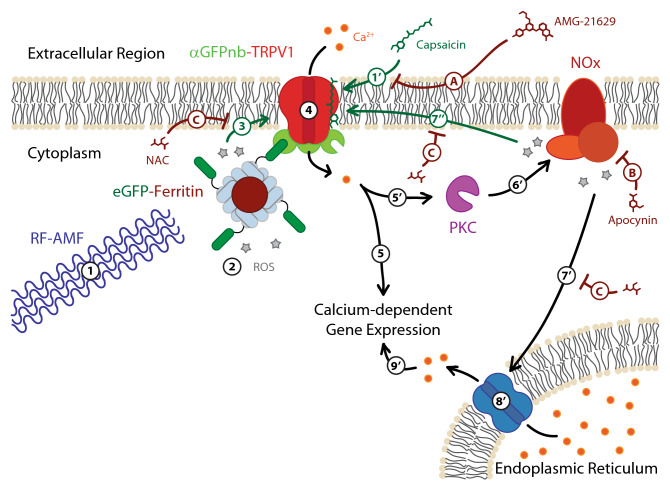
Schematic of proposed ROS involvement in RF-AMF induced activation of TRPV1-ferritin magnetogenetic platform. Exposure to RF-AMF (1) stimulates ROS production at the surface of the magnetic ferritin nanoparticle core (2), which due to colocalization by the eGFP-αGFPnb interaction (3) induces TRPV1 gating of Ca^2+^ ions into the cell (4) and the subsequent signaling cascade resulting in Ca^2+^-dependent gene expression (e.g., production of SEAP) (5). Capsaicin binds to TRPV1 (1′) to sensitize TRPV1 gating (1–3) leading to greater Ca^2+^ flux into the cell (4). The increased Ca^2+^ flux still stimulates Ca^2+^-dependent gene expression (5) and may activate PKC (5′), which activates NOx assembly resulting in additional ROS production (6′) and initiates a positive feedback loop to further stimulate TRPV1 (7″) and gate additional Ca^2+^ ion flux into the cell (4). The high ROS production by NOx can result in stimulation of the endoplasmic reticulum (7′) to release its stored Ca^2+^ supply into the cytoplasm (8′) that then further upregulates Ca^2+^-dependent gene expression (9′). The TRPV1 antagonist AMG-21629 competitively inhibits capsaicin binding to the TRPV1 tetramer complex (A). Apocynin inhibits NOx-catalyzed superoxide production (B). *N*-acetylcysteine (NAC) scavenges ROS (C).

Based on the combined roles of AMF and capsaicin, and the effects of the inhibitors tested, we posit that ROS contributes to at least one dimension of the mechanism of ferritin-mediated, AMF-induced TRPV1 activation and calcium signaling leading to SEAP production (Fig. 5). RF-AMF may generate a small amount of ROS at the surface of the magnetic ferritin nanoparticle core^32,33^ that, as a result of colocalization of ferritin to the TRPV1, induces or sensitizes TRPV1 gating of Ca^2+^ into the cell, thereby activating subsequent signaling cascades leading to Ca^2+^-dependent SEAP production. In the absence of capsaicin, ROS activation would be inhibited only by AMG-21629, as it binds directly to TRPV1. While a small, measurable inhibition of TRPV1 activation by AMF was observed upon addition of 5.0 mM NAC, due to the colocalization of the ferritin nanoparticles to the TRPV1 channels NAC-based ROS scavenging is likely inefficient and would require an excessively high concentration to fully inhibit AMF-induced gating. This inefficiency could also be compounded by the need for NAC to be biologically converted into a sulfane sulfur product in order to observe its ROS scavenging properties^48^.

As indicated above, capsaicin is known to induce a conformational change to TRPV1^40,41^ and such a conformational change may be facilitated by AMF exposure, potentially through ROS-based oxidative cysteine modification^25,49^, thereby lowering the temperature threshold of the channel. The combined effect of AMF-induced ROS generation and capsaicin would then result in greater Ca^2+^ flux into the cell than in the presence of AMF alone. This higher Ca^2+^ flux may, in turn, activate PKC^50^, which activates NOx membrane assembly through phosphorylation of p47^*phox*^ and leads to additional ROS production^34^. The addition of apocynin has the potential to further increase this ROS production^51^, however, when it is enzymatically-activated by conversion into a number of oligomeric species it becomes an inhibitor of p47^*phox*^ binding to membrane-associated p22^*phox*^^37,38^. Critically, HEK-293T cells have intracellular peroxidase-like enzymes^52^ and can therefore oxidize apocynin, thus reducing this additional ROS production and, consistent with the observed results, inhibit reporter expression. In the absence of apocynin, the higher levels of ROS can further stimulate TRPV1 gating of Ca^2+^ in a positive feedback loop while potentially also simultaneously stimulating the endoplasmic reticulum to release some of its stored Ca^2+^ supply into the cytoplasm^53^, both of which would further contribute to Ca^2+^-dependent gene expression. Inhibition of AMF-capsaicin potentiation of TRPV1 gating by NAC is consistent with the elimination of large amounts of ROS that may be generated by an activated NOx.

This ROS-based mechanism is supported by the recent work of Hernández-Morales et al. on the chemical activation of ferritin-based magnetogenetic platforms exposed to RF-AMF^54^. Activation of various TRP channels conjugated to ferritin by the kininogen-1 domain 5, a known ferritin-binding domain, was shown to increase Ca^2+^ influx upon exposure to 12 μT, 180 MHz AMF. They demonstrated that the RF-AMF increased the labile iron pool resulting in generation of ROS that subsequently increased the oxidized lipid content in the cell membrane^54^. These results support the proposed chemical mechanism that ROS generated by ferritin gates TRPV1 specifically when exposed to RF-AMFs. These data, however, do not exclude the possibility of a physical mechanism contributing to the gating of the TRP channels, as has also been suggested by previous experimental and theoretical studies, when using other magnetic stimulation methods such as static permanent magnets^55–57^ and direct current (DC) electromagnets^17,58^. For example, Duret et al. proposed an intriguing magnetocaloric mechanism for ferritin-driven ion channel activation^42^ that depends on ferritin’s superparamagnetic properties that can potentially enable channel opening without bulk heating^46^. Barbic performed theoretical modeling of ferritin and found that at high iron loading, clusters of iron spins can exist that generate heating above k_B_T in the presence of a sufficiently strong magnetic field^46^. This led to the suggestion that a second possible mechanism could be considered in which magnetic fields could gate TRPV1 tethered to ferritin through mechanical actuation, either by diamagnetic repulsion of the cell membrane by ferritin or as a result of an Einstein-de Haas effect^46^. In the latter case, a change in the angular momentum of a ferritin nanoparticle in an AMF translates into rotation of the ferritin nanoparticle, which can apply sufficient torque to gate an adjacent TRPV1 channel. Critical to understanding which of these potential physical and chemical mechanisms contribute to ferritin-mediated AMF-induced TRPV1 activation are the properties of the ferritin transceiver. To more conclusively model the ferritin-mediated mechanism, a better understanding of the role of in vitro cell culture conditions on ferritin’s precise magnetic state (e.g., superparamagnetic, paramagnetic, antiferromagnetic, etc.) and properties of its iron oxide core (e.g., composition, size, domain structure, crystallinity, clustering, etc.) will be required.

In conclusion, a sharp cutoff of channel activation occurs when the average field strength drops below a threshold of ~ 23 mT. Furthermore, AMF-induced gating of the ion channel is significantly enhanced by the presence of 1.0 μM capsaicin. Mechanistic evaluation of AMF-induced TRPV1 activation (alone or potentiated with capsaicin) indicates that ROS play a significant role in activating TRPV1, and inhibition of ROS production or scavenging of generated ROS leads to reduced TRPV1 activation. Such a chemical mechanism would be consistent with ferritin’s iron oxide nanoparticle structure and would not require specific physical mechanisms (e.g., localized heating or mechanical force) to gate TRPV1. While the combination of such physical mechanisms together with a ROS-based mechanism cannot be ruled out, the hypothesis that a ROS mechanism is directly involved in magnetogenetics enables further optimization of ion channel activation to advance synthetic biology in vitro and potential therapeutic applications clinically.

## Materials and methods

### Genetic constructs and cell lines

The αGFPnb-TRPV1-T2A-eGFP-FtD (Fig. 1a (i)) genetic construct was synthesized using the method outlined by Stanley et al*.*^15^. The Ca^2+^-dependent SEAP reporter (CaR-SEAP) was based on the Ca^2+^-dependent proinsulin reporter by Stanley et al*.*^15^. The proinsulin gene was removed from the construct between the Ca^2+^-dependent minimal promotion system and the SV40 poly(A) sequence using HindIII/NdeI restriction enzymes and replaced by a SEAP cDNA sequence. Human embryonic kidney (HEK-293T; ATCC, CRL-3216) cells were used for all studies. Stable clonal HEK-293T cell lines for each of the MSCV constructs were generated and selected for strong expression using fluorescence-activated cell sorting. The stable clonal cell lines for the full system (nbV1/FtD) and the precursor eGFP-FtD constructs are referred to as HEK-nbV1/FtD and HEK-FtD, respectively. For all cells, SEAP production was introduced using transient transfection of the CaR-SEAP construct.

### Ferritin immunoprecipitation

HEK-FtD cells were subcultured in 10-cm tissue culture dishes at low density (2–3 × 10^4^ cells∙cm^−2^) in 15 mL Dulbecco’s Modified Eagle Medium (DMEM; Gibco) supplemented with 10% fetal bovine serum (FBS; Gibco) at 37 °C in a humid 5% CO_2_ incubator. After 28–32 h, the medium was changed to DMEM supplemented with 1 or 10% FBS, 0 or 2 mg∙mL^−1^ holo-transferrin (HTF; Sigma-Aldrich), and 0 or 500 μM ferric citrate (FeC; Sigma-Aldrich). After 24 h, the cells were harvested for their ferritin using Tris-buffered saline (TBS)-based lysis buffer with protease inhibitors and no metal-ion chelators. Lysed cells were collected, agitated at 250 rpm for 30 min at 4 °C, and then centrifuged at 20,000x*g* and 4 °C for 20 min.

To purify eGFP-tagged chimeric ferritin, the FLAG-tag (DYKDDDDK) linker of the eGFP-FtD was used as an immunoprecipitation target. Monoclonal ANTI-FLAG M2 antibodies conjugated to agarose beads (αFLAG-beads; Sigma-Aldrich) were used for affinity capture of the FLAG-tagged ferritin following the Sigma recommended protocol with the noted exceptions. For binding, each lysate was agitated at 250 rpm on an oscillating shaker with αFLAG-beads slurry mixture for 3 h at 4 °C to capture αFLAG-tagged ferritin. Once bound, the column of αFLAG-beads was formed and washed three times with cold TBS. FLAG-tagged ferritin was eluted from the column using five washes of 200 μg∙mL^−1^ 3xFLAG peptide (APExBIO) in cold TBS. The eluted mixtures of αFLAG-tagged ferritin (> 480 kDa), unassembled eGFP-FtD (71.5 kDa), and excess 3xFLAG peptides (2.9 kDa) in TBS were then separated by 100 kDa molecular weight cut-off centrifugal filters (Millipore, centrifuged at 14,000x*g* for 15–20 min at 4 °C) to obtain a purified solution of FLAG-tagged ferritin. Samples were then resuspended in cold TBS, quantified using the Micro BCA protein assay (Pierce), and stored at 4 °C until characterized.

### Ferritin characterization

The purified eGFP-tagged ferritin was characterized by transmission electron microscopy (TEM) and high-resolution (HR) TEM (JEM-2100F, JEOL) to assess core size and crystal structure, respectively. Samples were buffer exchanged from TBS with deionized water to remove salt and diluted to 5–50 μg∙mL^−1^ of eGFP-ferritin protein. Diluted samples were then drop-cast onto copper grids and dried prior to TEM and HRTEM. To visualize and measure the size of the ferritin protein corona, negative staining using 1% (w/v) phosphotungstic acid was employed with TEM. TEM images were analyzed for core and protein size using the ImageJ FIJI (National Institute of Health) particle analysis function. HRTEM images were analyzed using the fast Fourier transform (FFT) function in ImageJ FIJI to generate electron diffraction (ED) patterns.

### AMF stimulation system

A W-5/500 power station with HS-8 heat station was used with a custom 2-turn induction coil (*L* ≈ 0.34 μH), interchangeable capacitors, and adjustable transformer to generate AMFs of ~ 350–500 kHz (UltraFlex Power Technologies). A custom insulated sample holder was constructed to hold four samples within the same volumetric positions of the coil across all experiments (see Supplementary Fig. 3a). Capacitances of 0.3, 0.4, 0.5, and 0.6 μF were used to generate frequencies of 500–502, 435–436, 387–388, and 353–354 kHz, respectively. Heat generation was regulated by passing cold water through the hollow tube of the induction coil. The temperature for each AMF stimulation condition was determined by pre-warming the coil for 60–90 min and then periodically measuring the temperature of the treatment buffer within each insulated holder until equilibrium was reached. A non-CO_2_ incubator was set to the coil’s equilibrium temperature to create the corresponding AMF− temperature control condition.

### AMF stimulation, potentiation, and inhibition studies

HEK-293T and HEK-nbV1/FtD cells were cultured in DMEM supplemented with 10% FBS in T75 flasks at 37 °C in a humid 5% CO_2_ incubator (same for all steps unless otherwise noted). HEK-nbV1/FtD cells were subcultured onto 12-mm-diameter No. 2 cover glass slips in 24-well plates. All cover slips were pretreated with fibronectin (Sigma-Aldrich; 10 μg∙mL^−1^ in DPBS) to enhance cell adhesion. Cells were plated to the cover glass slips at low density (~ 2.1 × 10^4^ cells∙cm^−2^) in 500 μL of DMEM supplemented with 10% FBS. After 28–30 h, HEK-nbV1/FtD cells were transfected with the CaR-SEAP construct (248 ng∙well^−1^) using Lipofectamine 2000 (2.5 ng:1 ng DNA; Invitrogen) in Opti-MEM (OMEM; Gibco) supplemented with 2 mg∙mL^−1^ HTF following a standard Lipofectamine 2000 transfection protocol. Similarly, HEK-293T cells were transfected with a 1:2 molar ratio of the nbV1/FtD construct (252 ng∙well^−1^) to the CaR-SEAP construct (248 ng∙well^−1^) following the same Lipofectamine 2000 protocol. The medium was changed 16–18 h post-transfection to 500 μL OMEM supplemented with 1% FBS and 500 μM FeC. After 8 h growth at 37 °C, the temperature was reduced to 32 °C for 16–18 h in preparation for AMF stimulation.

Cells on cover slips were transferred to 400 μL of Ca^2+^-enriched (using CaCl_2_; Sigma-Aldrich) OMEM (base Ca^2+^ concentration of 0.9 mM) to a final concentration of 2.5 mM (Ca-OMEM). AMF-agonist potentiation experiments used 1.0 μM capsaicin (Tocris) in Ca-OMEM. For AMF inhibition studies, Ca-OMEM was supplemented with either 100 nM AMG-21629 (3-amino-5-[[2-[(2-methoxyethyl)amino]-6-[4-(trifluoromethyl)phenyl]-4-pyrimidinyl]oxy]-2(1*H*)-quinoxalinone; Tocris), 100 μM apocynin (4-hydroxy-3-methoxyacetophenone; Sigma-Aldrich), or 5.0 mM *N*-acetylcysteine (NAC; Sigma-Aldrich). Combinatorial medium conditions were created between capsaicin and each of the inhibitors (Inhib) to create four conditions to study AMF activation: basal (Cap−/Inhib−), potentiation (Cap+/Inhib−), inhibitory (Cap−/Inhib+), and inhibited potentiation (Cap+/Inhib+). HEK-nbV1/FtD cells were used for all AMF studies except the apocynin and NAC inhibition studies, which used HEK-293T cells transiently expressing the nbV1/FtD platform.

The insulated sample holder was then secured within the coil, covered, and exposed to continuous AMF at set field strengths (controlled by applied current) and frequencies (set by capacitance) for 15–120 min depending on experimental condition. The AMF− controls were treated in an identical insulated sample holder with 400 μL of supplemented Ca-OMEM in the non-CO_2_ incubator at the coil’s equilibrium temperature. Post-treatment, cover slips were transferred back to 500 μL of DMEM with 10% FBS, incubated for 2 h (at 32 °C in a 5% CO_2_ incubator), and assayed with 3-(4,5-Dimethyl-2-thiazolyl)-2,5-diphenyl-2H-tetrazolium bromide (MTT; Sigma-Aldrich) using a standard MTT assay to determine cell number. The Ca-OMEM supernatant was collected, centrifuged to remove any cell matter, and stored at 4 °C until assayed for SEAP production. SEAP production was assayed through its conversion kinetics of *para*-nitrophenyl phosphate (pNPP; Sigma-Aldrich; 1 mg∙mL^−1^) to *para*-nitrophenol (pNP) in 1 M diethanolamine buffer (SeraCare; pH 9.8) by plate reader absorbance measurements at 405 nm. Each biological replicate was assayed in triplicate, compared to alkaline phosphatase (AP; Roche) standards to convert absorbance at 405 nm to SEAP concentration (mU∙mL^-1^), and then normalized to cell number determined by MTT assay (mU SEAP per 10^6^ cells).

### Statistics

All statistics were calculated using GraphPad Prism 7 software. Data was first checked for outliers using the robust regression and outlier removal (ROUT) method with a false discovery rate set by Q = 1%. After removing outliers, all data was tested for Gaussian distribution and variance using either the D’Agostino-Pearson omnibus normality test (*n* ≥ 8) or the Shapiro–Wilk normality test (*n* < 8). For one interaction comparisons, statistical significance of normally distributed data was calculated using one-way ANOVA with Šídák’s multiple comparisons test while for non-normally distributed data the Kruskal–Wallis nonparametric test with Dunn’s multiple comparisons test was used. For multiple interaction comparisons, either two-way or three-way ANOVAs with Tukey’s multiple comparisons test was used to calculate significance for both normally and non-normally distributed data. Statistical significance for all multiple comparisons tests was determined using a two-tailed, family-wise significance of 0.05 (95% confidence interval). Statistical analysis method, data presentation, total number of samples in each group (*n*), and *p*-values are as indicated on each figure and/or within each figure legend.

### Ethics statement

All methods were carried out in accordance with relevant guidelines and regulations. No animals were directly involved in the study.

## Supplementary information

Supplementary information

## Data Availability

The data sets generated during and that support the findings of this study are available from the corresponding author upon reasonable request.
