# Supplementary material for: Uncovering a possible role of reactive oxygen species in magnetogenetics
Source: Sci Rep. 2020 Aug 4;10:13096. doi: 10.1038/s41598-020-70067-1 (PMC7403421; doi:10.1038/s41598-020-70067-1)
Supplement: Supplementary file 1 — Supplementary information [file 41598_2020_70067_MOESM1_ESM.docx]

**Supplementary Information for**

**Uncovering a Possible Role of Reactive Oxygen Species in Magnetogenetics**

Matthew I. Brier^1^, Jordan W. Mundell^1^, Xiaofei Yu^2†^, Lichao Su^3^, Alexander Holmann^1^, Jessica Squeri^1^, Baolin Zhang^3^, Sarah A. Stanley^4^, Jeffrey M. Friedman^2,5^, & Jonathan S. Dordick^1,6^*

^1^Department of Chemical and Biological Engineering, and Center for Biotechnology & Interdisciplinary Studies, Rensselaer Polytechnic Institute, Troy, NY 12180

^2^Laboratory of Molecular Genetics, Rockefeller University, New York, New York 10065

^3^State Key Laboratory Breeding Base of Nonferrous Metals and Specific Materials Processing, College of Material Science and Engineering, Guilin University of Technology, Jian Gan Road 12, Guilin 541004, China

^4^Diabetes, Obesity and Metabolism Institute, Icahn School of Medicine at Mount Sinai, New York, NY 10029

^5^Howard Hughes Medical Institute, New York, NY 10065

^6^Departments of Biomedical Engineering and Biological Sciences, Rensselaer Polytechnic Institute, Troy, NY 12180

^†^Present address: State Key Laboratory of Genetic Engineering, School of Life Sciences, Fudan University, Shanghai 200438, China

Verification of nbV1/FtD construct expression and response to capsaicin

While expression and localization of the nbV1/FtD construct were weakly visible in stable cells, transiently transfected cells that overexpressed the TRPV1 and eGFP-tagged ferritin showed much clearer membrane localization. Supplementary Fig. 1 clearly shows this overexpression due to transient transfection and how the construct is found in the cell membrane of positively transfected cells.


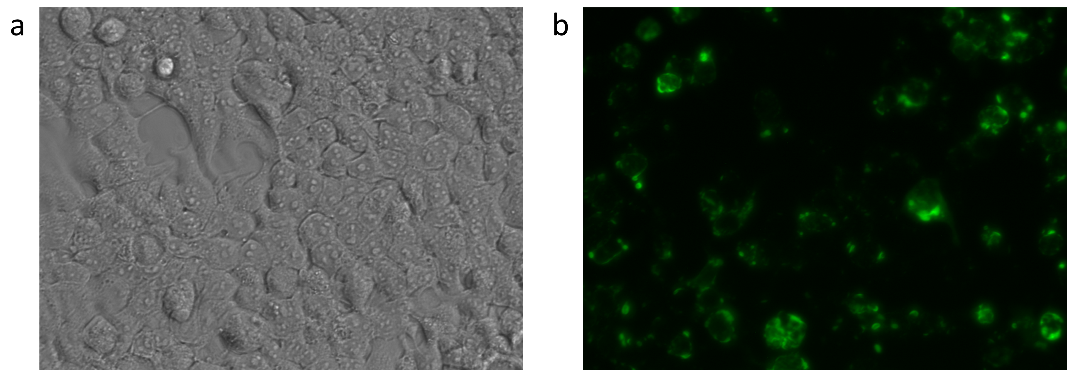


**Supplementary Figure 1.** **Transient expression of magnetogenetic platform in HEK-293T cells.** (**a**) Brightfield and (**b**) fluorescent images of transient expression of nbV1/FtD in HEK-293T cells, showing enhanced expression and clear localization of the αGFPnb-TRPV1-T2A-eGFP-FtD construct (**Fig. 1a, i**) to cell membranes.

Normal function of the TRPV1 channel in the membrane of HEK-nbV1/FtD cells was tested by live cell calcium imaging using Fluo-4 AM fluorescent calcium dye (Invitrogen, cell permeant version). HEK-nbV1/FtD cells were subcultured onto 12-mm cover glass slips in 24-microwell plates at ~1.0×10^4^ cells∙cm^-2^ in 500 μL of DMEM supplemented with 10% FBS, 2 mg⋅mL^-1^ HTF, and 500 μM FeC and grown at 37°C in a 5% CO_2_ incubator for 36 h before being transitioned to 32°C for an additional 12 h before beginning calcium imaging. For imaging, cells were loaded with calcium-binding Fluo-4 AM green fluorescent dye using 500 μL Fluo-4 AM permeation buffer (3 μM Fluo-4 AM dye; 200 μM (±)-sulfinpyrazone, Sigma-Aldrich; 0.02% Pluronic F-127, Invitrogen; in DPBS) during a 30 min incubation at room temperature and shielded from light. Cells were then incubated 500 μL of PBS with 200 μM (±)-sulfinpyrazone for 20 min at room temperature and shielded from light. Imaging was performed in the center of No. 1.5 glass-bottom 35-mm microscopy dishes (MatTek Corporation) with 1000 μL Ca-OMEM using a Nikon Eclipse TE200 epifluorescence microscope with connected halogen lamp (Chiu Technical Corporation Mercury-100W) and camera (SPOT Insight QE digital camera, Diagnostic Instruments, Inc.) using the 20x objective (Nikon S Plan Fluor ELWD). Samples were imaged for 6 min with images taken every 2-5 s. The first 2 min were used to capture baseline cell fluorescence after which capsaicin was added to adjust the concentration to 10 μM and induce activation of the nbV1/FtD platform. Supplemental Fig. 2 shows agonist-induced TRPV1 gating of Ca^2+^ flux into HEK-nbV1/FtD cells in response to 10 μM capsaicin treatment.


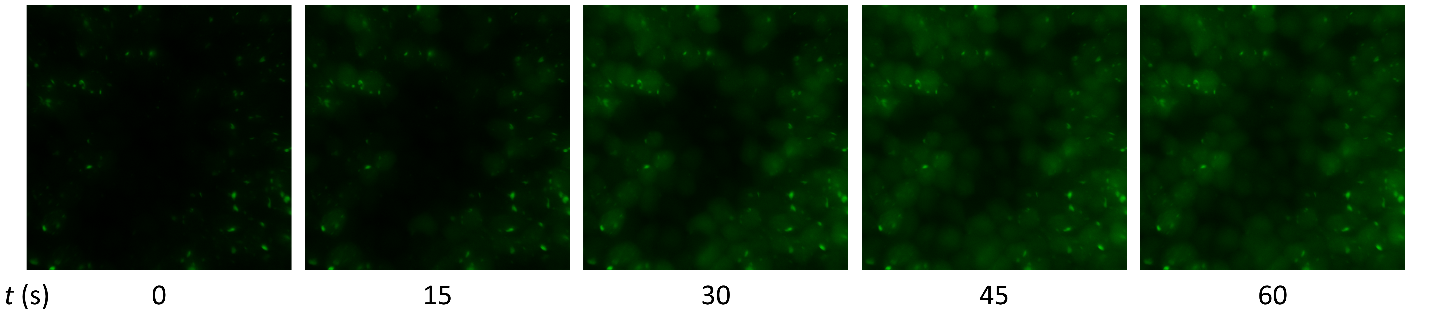


**Supplementary Figure 2. Capsaicin induced calcium flux into HEK-nbV1/FtD cells.** Fluorescent images of HEK-293T cells stably expressing the HEK-nbV1/FtD construct gating calcium into the cell, visualized by green fluorescence of Fluo-4 AM calcium dye, in response to 10 μM capsaicin dose introduced at *t* = 0 s. Onset of Fluo-4 AM fluorescence was visible at *t* = 15 s and continued until plateauing at *t* = 60 s.

Development of custom device for generating variable magnetic fields

In prior studies, AMFs at frequencies of 465-470 kHz and field strengths of 29-32 mT induced ferritin-mediated gating of TRPV1 assayed *in vitro* and *in vivo*. However, these studies employed a single activation condition and the effect of varying both magnetic field strength and frequency on TRPV1 gating was not evaluated. To vary both frequency and field strength, a custom RF induction system with a 2-turn coil was used. This enabled us to vary frequency from approximately 350 to 500 kHz and the associated magnetic field strength from approximately 5 to 34 mT.

Modeling of AMF strength generated by 2-turn induction coil

To understand the field strength parameter of radio frequency (RF)-generated alternating magnetic field (AMF) stimulation, it was necessary to examine the magnetic field generated within the induction coil by the RF induction system (UltraFlex Power Technologies, Ronkonkoma, NY), particularly because the field strength varies substantially with position inside the coil, and across each individual sample in its microwell holder. Thus, a Matlab program was written using the Biot–Savart Law (1), to calculate the magnetic field $\mathbf{B}$ created at position $\mathbf{r}$ in and around a coil with a given coil geometry $C$.

$$\mathbf{B}\left( \mathbf{r} \right)= \frac{\mu_{0}}{4\pi}\int_{C} \frac{Id\mathcal{l}\times\mathbf{r}^{\mathbf{'}}}{\left| \mathbf{r}^{\mathbf{'}} \right|^{3}}, \left( \text{1} \right)$$

To reasonably capture the topography of the magnetic field, the space around the 2-turn induction coil used in this work, was defined as a discrete mesh of points with positions $\mathbf{r}$ spaced at 0.5 mm increments in the $x$, $y$, and $z$ dimensions (~3.3×10^6^ points in total). For modeling purposes, a number of simplifying assumptions were made about the coil geometry, particularly that: (i) the turns of the coil could be modeled as concentric, stacked loops rather than a continuous, pitched spiral, and (ii) the coil’s surface could be modeled as a discrete mesh of points $\mathcal{l}$ (1.0×10^4^ points per turn) with the applied current uniformly distributed across the surface of the coil (100 points per cross-sectional slice of each turn). In accordance with this, the displacement vector $\mathbf{r}^{\mathbf{'}}$ from each spatial position $\mathbf{r}$ to each coil element $d\mathcal{l}$ was defined as $\mathbf{r}^{\mathbf{'}}\mathbf{=r}\mathcal{-l}$. Additionally, because the magnetic field generated by a coil is linearly proportional to the applied current $I$, as per the Biot–Savart Law, all modeling was performed under assumption that 1 A of alternating current was delivered from the power station to the system’s internal transformer (configured with a 25:1 ratio) where it was stepped up to 25 A and delivered to the coil (i.e.,$I \text{= 25 A}$). Thus, all values generated by the model were simply multiplied by the power station output displayed on the UltraFlex system control panel to determine field strengths during treatment.

The results of the modeling were depicted as color maps of the magnetic field components (defined in Cartesian coordinates as $B_{x}$,$B_{y}$, and $B_{z}$) for a number of cross-sectional slices of the space around the coil focused around the sample positions. Supplementary Fig. 3a depicts one such vertical, radially symmetric cross-section for $B_{z}$, the dominant component of the magnetic field strength within the coil as per contribution to the magnitude $\left| \mathbf{B} \right|$, as a function of position $\mathbf{r}$. Supplementary Fig. 3b correlates the color map to the absolute value of $B_{z}$ with respect to coil radius $r$ for a number of traces along fixed axial ($z$) positions, with the black 0.00 cm trace crossing through the center of the coil and samples. As per the 0.00, 0.25, and 0.55 cm curves (black, blue, and green, respectively), which are all within the volume of the coil, the dependence on $r$ for $B_{z}$ across the sample holders is more clearly evident than just from the color map in Supplementary Fig. 3a. Outside the volume of coil (red and cyan curves at 1.10 and 1.70 cm, respectively), the $B_{z}$ shows less dependence on $r$, however this does not factor in the curvature of magnetic flux lines and the corresponding increases in the $B_{x}$ and $B_{y}$ components. Supplementary Fig. 3c shows similar traces for $B_{z}$ but with respect to axial position $z$ for select radial positions and highlights the differential from the central region of the coil ($r=$ 0.00 cm, black curve) to the outer edge of the sample holder ($r=$ 1.70 cm, cyan curve) in both magnitude of $B_{z}$ and its coverage of the sample holder.


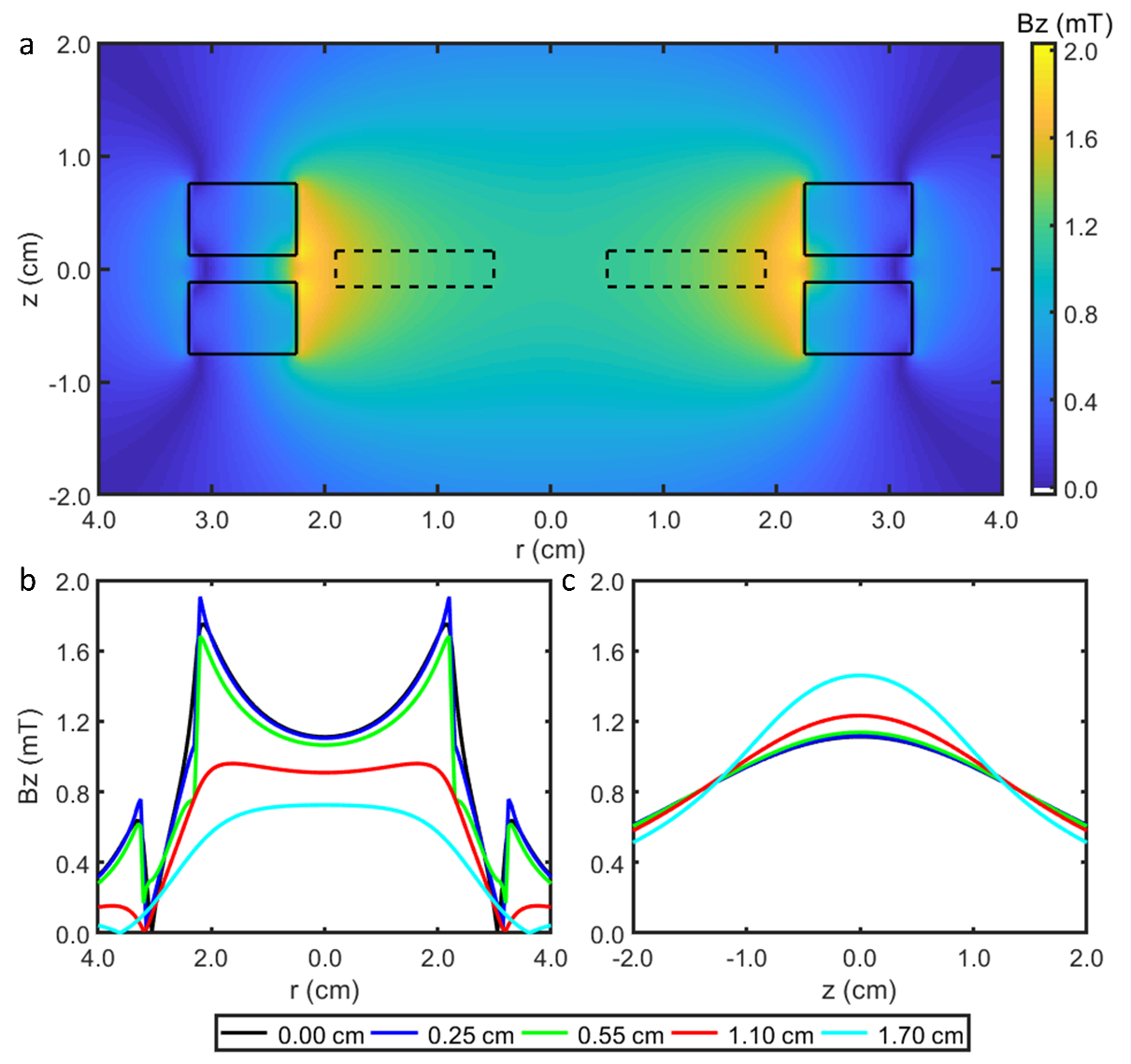


**Supplementary Figure 3.** **Biot–Savart Law modeling of RF-AMF created by the 2-turn coil system.** (**a**) Color map displaying magnetic flux along the *z*-axis, $B_{z}$, of RF-AMF created by the 2-turn coil used to study frequency dependence of the magnetic field. Heat map was normalized to 1 A of applied current by the power station, corresponding to 25 A at the coil based on the operating configuration of the system. Solid line rectangles represent the cross-section of the 2 turns of the coil, and the dashed line rectangles represent the approximate vertical and radial positions occupied by samples during RF-AMF stimulation. (**b**) Plot of $B_{z}$ with respect to radial position $r$ for select axial positions away from the center horizontal plane of coil. (**c**) Plot of $B_{z}$ with respect to axial position $z$ for select radial positions as measured from the central vertical axis of the coil. For both plot sets, only one direction was explored due to the symmetries present within the cylindrical coil.

To translate this model to the field gradient actually experienced by cell samples on cover slips within the coil during experimentation, all points $\mathbf{r}$ and their associated $\mathbf{B}$ from the discrete spatial mesh within the approximated volume of the four sample holder positions were extracted from the total data set. The relevant volume of each sample holder, where the cover slips could experience the RF-AMF stimulation, was defined in the $x$ and $y$ (radial) dimensions by the center point of each holders and the each holder’s inner diameter (~14 mm), not the cover slip diameter (~12 mm) as it could reside anywhere within the holder area during treatment. In the $z$ (axial) dimension, more leeway was required as fabrication of each of the individual sample holders varied (i.e., slight discrepancies in the thickness/height of the base support where the cover slip sits in the holder). Based on caliper measurements of all holders, there was a ~3.2 mm range centered on the central horizontal plane of the coil within which the cover slip could sit. These parameters together (visualized as in the vertical cross-section of Supplementary Fig. 3a) defined the positions within the coil where points were extracted (radial cross-section shown in Supplementary Fig. 4a).

From these extracted points, the components of the magnetic field, $B_{x}$,$B_{y}$, and $B_{z}$, and their magnitude, $\left| \mathbf{B} \right|$, were plotted to understand the combined distributions within the four holders. Given the use of Cartesian coordinates, the non-dominant $B_{x}$ and $B_{y}$ components when taking all four holders into account, generated identical, symmetric distributions (Supplementary Fig. 4b; noted as $B_{r}$ to represent either component, not the cylindrical coordinate radial component of $\mathbf{B}$, $B_{r}$). Supplementary Fig. 4c shows the distribution in the dominant $z$-component (along the axial direction of the coil) of the magnetic field, which shares an almost identical value and distribution to the magnitude $\left| \mathbf{B} \right|$ in Supplementary Fig. 4d. From the strong skew towards the lower field strengths in Supplementary Fig. 4c,d, it is clear that the average field strength, ~1.31 mT, is dominated by the greater proportion of the sample volume towards the inner region of the coil. While the distribution of field strengths is clearly weighted towards the lower field strengths, it does range from a minimum field strength of ~1.14 mT to a maximum of ~1.61 mT. Beyond defining the actual RF-AMF stimulation strength experienced by the cells, this variation in field strength across the sample could suggest that magnetogenetic controlled gene expression is only observed when a sufficient percentage of cells within the sample is within the region of the field with strength equal to or exceeding the threshold for stimulation.


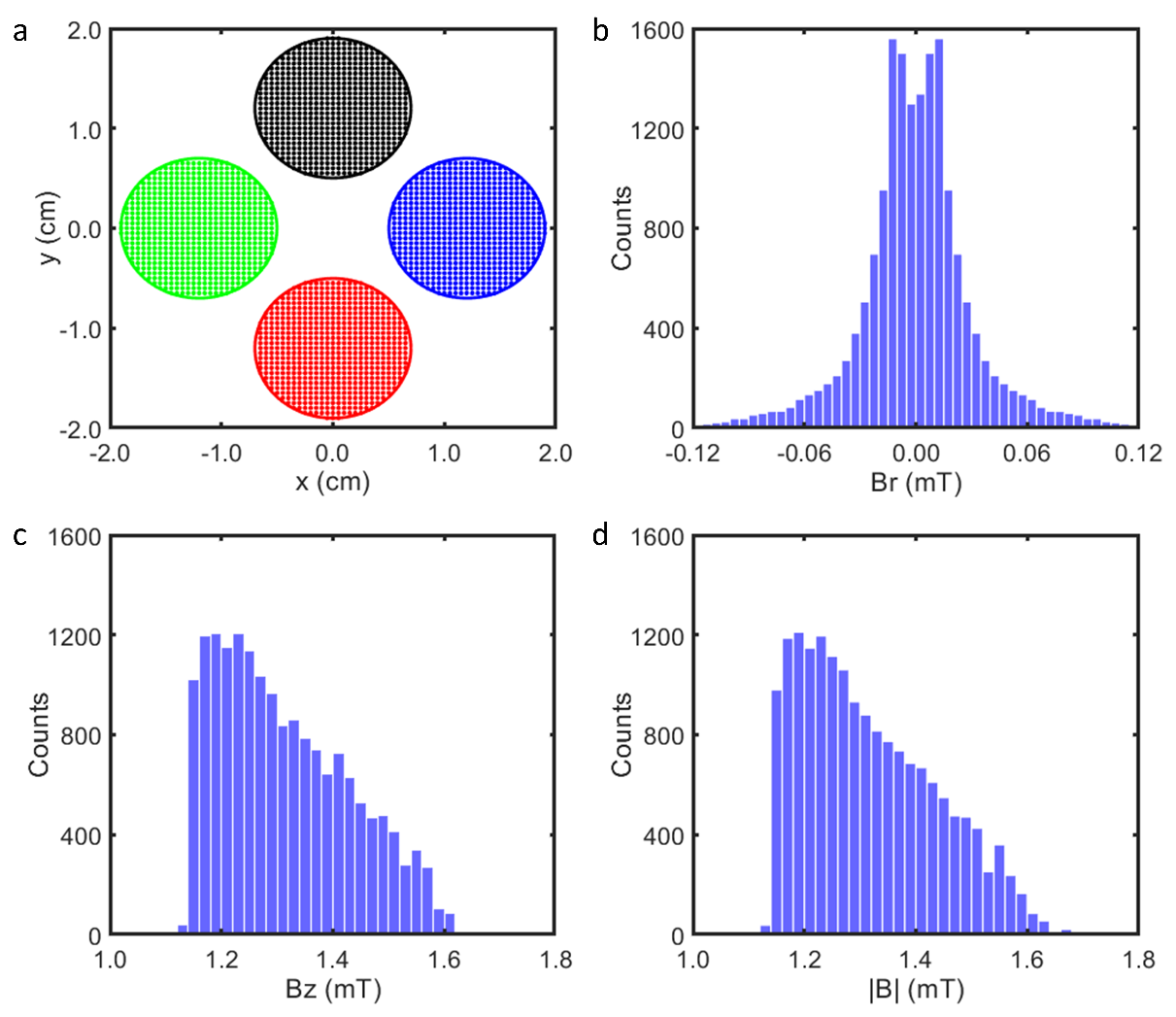


**Supplementary Figure 4.** **Determination of RF-AMF strength within sample holders in the 2-turn coil.** (**a**) Diagram of horizontal cross-section of four sample holder positions within 2-turn coil and points marking the sampling mesh for magnetic field strength within the holders at 0.5 mm increments. (**b-d**) Histograms depicting the distributions of field strength at each point within the sample holders as the (**b**) $x$- and $y$-components (radial) of the flux, $B_{x}$ and $B_{y}$ respectively, which due to symmetry are noted as $B_{r}$, (**c**) $z$-component (axial) of the flux, $B_{z}$, and (**d**) the magnitude of the magnetic flux, $\left| \mathbf{B} \right|$. The sign of $B_{r}$ and $B_{z}$ values in the distribution denote the direction of that component in the $\mathbf{B}$ vector, that is negative $B_{r}$ values are that magnitude in the negative $x$ or $y$ direction. Given the histograms in (**c,d**), it is clear the field strength is dominated by the axial component, $B_{z}$.

As a final model validation, and to ensure that the model assumptions did not compromise its efficacy of predicting the field strength experienced by samples, the field strength at the center of one of the sample holders was measured and compared to the model. This was done by measuring the electromotive force (EMF), $\mathcal{E}$, induced in a probe coil and converting it to the magnetic field strength based on Faraday’s and Lenz’s Laws (2 and 3),

$$\mathcal{E= -}N_{p}\frac{d\Phi_{B}}{dt}, \left( \text{2} \right)$$

where $N_{p}$ is the number of turns of the probe coil and $\frac{d\Phi_{B}}{dt}$ is the change in magnetic flux, $\Phi_{B}$, over time observed by the probe coil. The magnetic flux is defined as

$$\Phi_{B}=\mathbf{B}\cdot\mathbf{A}=B_{z}A_{p}\cos\left( \omega t \right) \left( \text{3} \right)$$

where the key measured component of the magnetic field $\mathbf{B}$ is the dominant axial component $B_{z}$ through the cross-sectional area of the probe coil $A_{p}$ that is normal to $B_{z}$. As the RF-AMF is oscillating, $\cos\left( \omega t \right)$ denotes the waveform with angular velocity $\omega$, which correlates to AMF frequency $f$ by the relationship $\omega=2\pi f$. Equations (2) and (3) together allow for the measurement of magnetic field as a function of EMF over time and critically, when $\cos\left( \omega t \right)=1$, the peak magnetic field in the axial direction, $B_{z,\max}$, when axial directions of the probe coil and the RF induction coil are parallel to each other. The peak magnetic field experienced per oscillation was calculated using the measured maximum EMF $\mathcal{E}_{\max}$ using the conversion

$$B_{z,\max}=\frac{\mathcal{E}_{\max}}{2\pi fN_{p}A_{p}}. \left( \text{4} \right)$$

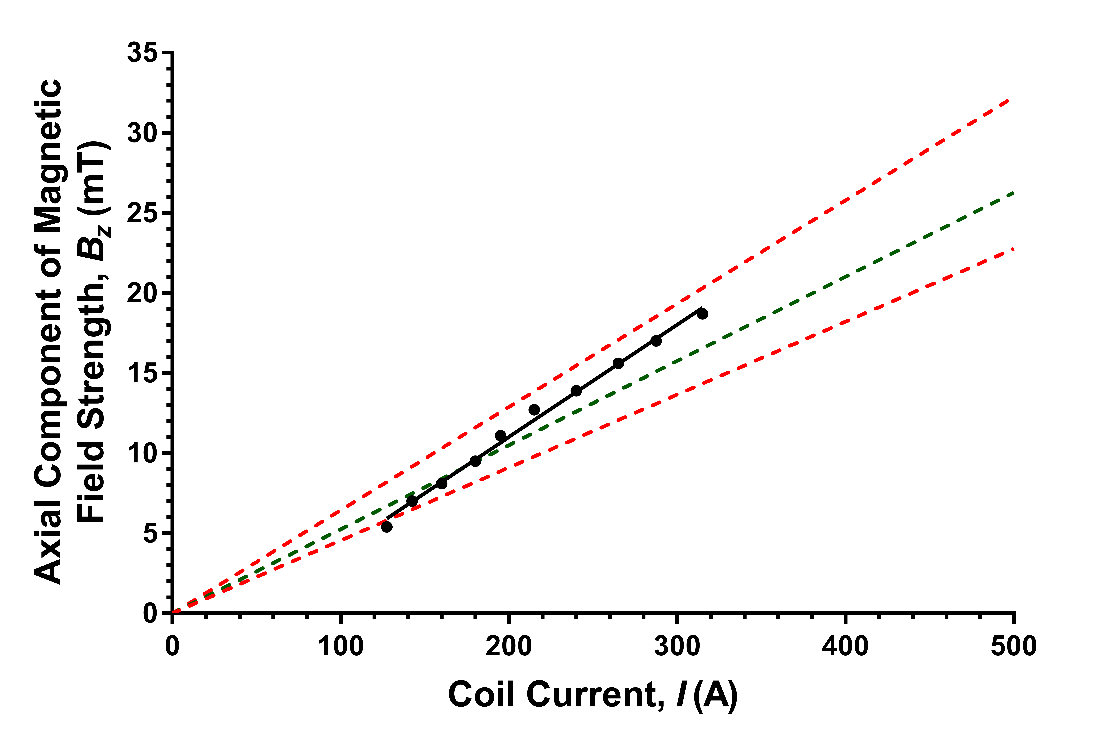


**Supplementary Figure 5.** **Correlation between Biot–Savart Law modeling of the 2-turn coil and probe sampling.** Measurements of axial component of field strength, $B_{z}$, made by small 10-turn solenoid probe placed in the center of one of the four sample positions (black dots). Each point marks a 5% increase power output by the system when running at 500 kHz. The dashed lines display $B_{z}$ as calculated by the Biot–Savart Law modeling of the 2-turn coil with the green line marking the mean and the red lines marking the minimum and maximum, respectively, possible field strengths a sample experience.

**Translation of AMF strength modeling to *in vitro* treatment conditions**

The heat map and positional plotting of magnetic flux (i.e., field strength) in Supplementary Fig. 3 distinctly show the variability in stimulation strength within the coil. Considering the central horizontal plane of the 2-turn coil (Supplementary Fig. 3b) where the magnetic flux ranges from 1.11-1.75 mT (black line) in terms of the average operating current of 495 A for the frequency studies, the range is equivalent to 22.0-34.7 mT. This disparity from the coil center (22.0 mT) to the coil edge (34.7 mT) is significant, particularly when considering that a field strength of 29-32 mT was previously required to show Ca^2+^ influx. Thus, substantial regions of the coil appear to experience field strengths significantly below the threshold for stimulation while others experience a greater field strength. Supplementary Fig. 3c shows this same variation in field strength in terms of vertical position at a number of radial distances away from the central vertical axis.

Taking into account modeling of the sample geometry in Supplementary Fig. 4, the distribution in field strengths experienced by cells on an individual cover slip becomes more apparent. That is, discounting the radial dimension (Supplementary Fig. 4b) due to how small it is compared to the axial dimension (Supplementary Fig. 4c) in the solenoid coil configuration we used, the average flux in Supplementary Fig. 4d of ~1.32 mT translates to ~26.1 mT at 495 A. This means that while cells can experience higher field strengths closer to the coil edge, the majority of cells experience stimulation well below that upper limit (~31.9 mT at 495 A). As the field strength decays slower the further it is from the coil edge, the majority of cells experience the field at strengths (~22.5 mT at 495 A) closer to the center of the coil (~22.0 mT). Validation of the modeling using the custom 10-turn coil probe (Supplementary Fig. 5) only serves to further emphasize this disparity, particularly as the probe was placed in the center of a treatment well to take an average measurement over its cross-sectional area. As such, all treatment conditions in the body of the work are discussed using the average magnetic field strength samples can experience at a given RF-AMF treatment setting and not based on the upper limit defined by the coil.

**Inhibitors of magnetogenetics**

Inhibition of capsaicin activation of the nbV1/FtD magnetogenetic platform, as measured by SEAP expression, was first explored in 96-well plate format to assess the dose dependence of both capsaicin and the inhibitor on the observed response. A protocol similar to the 12-mm cover slip experiments in 24-well plates was used. HEK-nbV1/FtD cells maintained in T75 flasks were subcultured into 96-well plates pretreated with fibronectin (10 μg⋅mL^-1^ in DPBS) to enhance cell adhesion. Cells were plated to 96-well plates at low density (2.6×10^4^ cells⋅cm^-2^) in 100 μL of DMEM supplemented with 10% FBS and grown at 37°C in a 5% CO_2_ incubator. After 28-30 h, HEK-nbV1/FtD cells were transfected with the CaR-SEAP construct (87 ng⋅well^-1^) using Lipofectamine 2000 (2.5 ng:1 ng DNA) following the 96-well plate version of the same Lipofectamine 2000 transfection protocol with OMEM supplemented with 2 mg⋅mL^-1^ HTF. At 16-18 h following transfection, cells were checked for growth and media was changed to 100 μL OMEM supplemented with 1% FBS and 500 μM FeC. After media change, cells were incubated at 37°C and 5% CO_2_ for ~24 h. Unlike with the cover slip protocol, which includes the temperature reduction to 32°C for 16-18 h to prepare for the lower temperatures of the AMF studies, there is no temperature reduction as the goal for these experiments was to observe combinatorial capsaicin-inhibitor treatments under optimal 37°C conditions.

After incubation for 24 h in reduced serum to decrease background SEAP production, samples were incubated in 100 μL of Ca-OMEM (2.5 mM Ca^2+^) supplemented with capsaicin-inhibitor combinations. Stocks of AMG-21629 (1000 nM), apocynin (500 μM), and NAC (10 mM) were made and then serial diluted to make at total of seven concentrations and a 0 M control for each inhibitor. A portion of each of these solutions was then independently used to generate a capsaicin (5.0 μM) solution followed by serial dilution to prepare five capsaicin concentrations and a no agonist control. HEK-nbV1/FtD cells in the 96-well plates were incubated with their respective capsaicin-inhibitor mixtures for 4 h in an incubator at 37°C and 5.0% CO_2_. After incubation was complete, the supernatant was collected in U-bottom 96-well plates, centrifuged, and stored at 4°C until assayed for SEAP production. The cells were then incubated in 100 μL of DMEM supplemented with 10% FBS for 2 h and then assayed for cell number using the standard MTT protocol scaled to 96-well plate format. The cell supernatant was assayed for SEAP production by kinetics of pNPP conversion to pNP by measuring absorbance at 405 nm, standardized against AP (mU∙mL^-1^), and then normalized to cell number (mU SEAP per 10^6^ cells).

The results of these combinatorial dose response tests are shown in Supplementary Fig. 6a,c,e for AMG-21629, apocynin, and NAC, respectively. During analysis, all inhibitor concentrations were converted to baseline values by subtracting out their respective no (0 μM) capsaicin samples (denoted in Supplementary Fig. 6 as $\left. {\Delta P}_{i,j} \right|_{C-0\mu M}$ with the subscripts indicating capsaicin and inhibitor concentrations, respectively). This was applicable to all concentrations of each inhibitor studied without skewing the trends in the data, as the 0 μM capsaicin dose yielded negligible SEAP response. Due to variations in response by the different capsaicin-inhibitor mixtures, particularly with the low doses of apocynin and NAC resulting in enhanced SEAP production under optimal capsaicin dose (0.50 and 1.0 μM for apocynin and NAC tests, respectively), the data was normalized to the peak stimulation condition without inhibitor (0 M) for each group (displayed as gray dashed lines in Supplementary Fig. 6a,c,e). The values for the 0.50 and 1.0 μM capsaicin doses (blue and red, respectively) were extracted for each inhibitor and plotted as inhibition curves in Supplementary Fig. 6b,d,f for AMG-21629, apocynin, and NAC, respectively.

For the inhibition curves at fixed 0.50 and 1.0 μM capsaicin, the half maximal inhibitor concentrations (IC_50_) were calculated using the continuous portions of the curves that fell below the normalized no inhibitor value (black dashed line in each plot) in a GraphPad Prism three parameter inhibitor versus response model where the bottom and top parameters were constrained to 0 and 1, respectively. Values over the no inhibitor concentration were not considered for calculating the inhibition curves and IC_50_ values because the interaction of optimal agonist dose with low levels of apocynin and NAC appear to activate TRPV1 response, perhaps through a cellular mechanism not related to TRPV1. Indeed, it has been shown that when excessive capsaicin is used to stimulate TRPV1 the channel can undergo endocytosis and is removed from the cell membrane, thereby resulting in reduced SEAP production observed at 5.0 μM capsaicin with no inhibitor present.


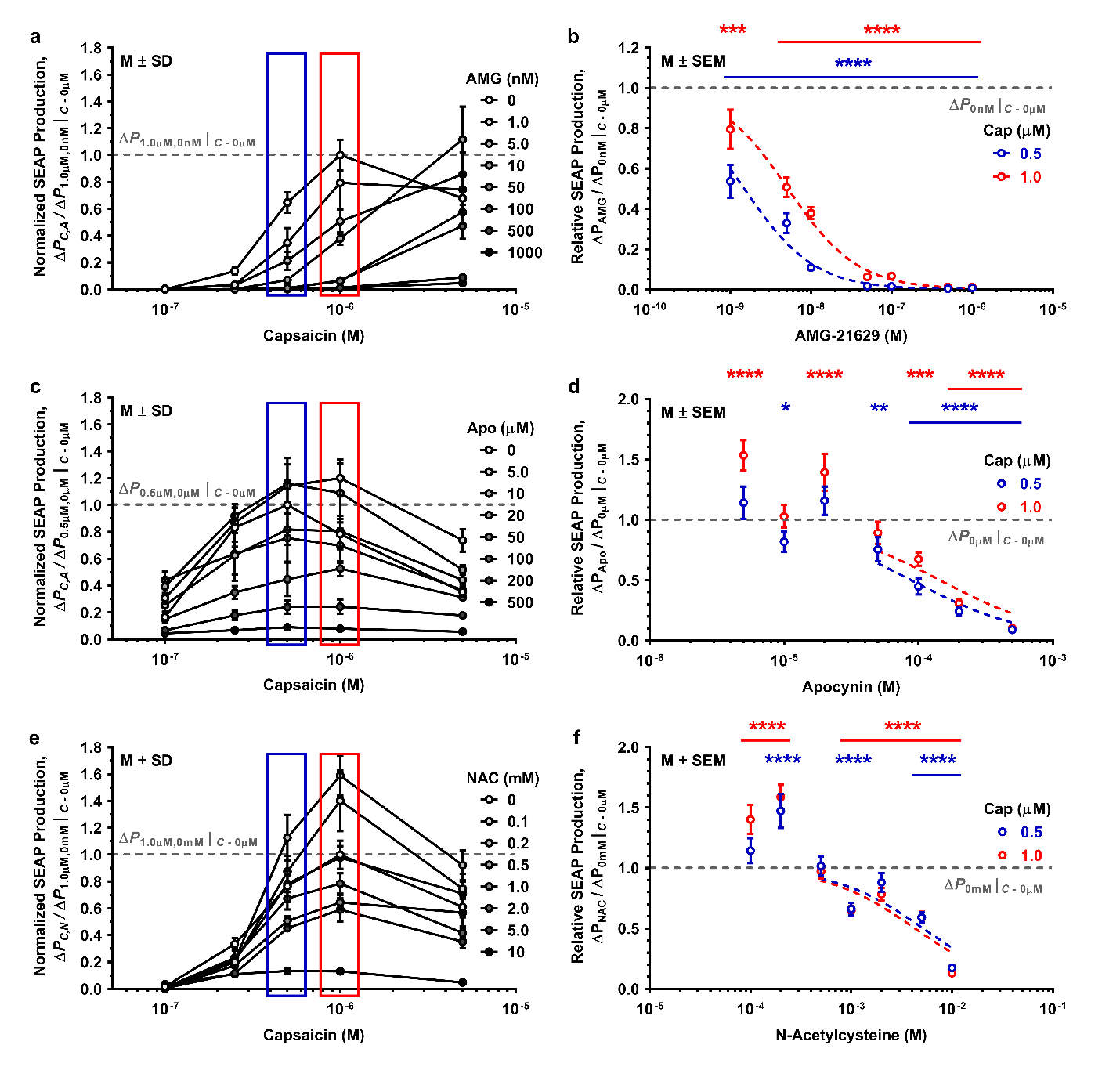


**Supplementary Figure 6. Magnetogenetic platform response to combinatorial stimulation by capsaicin and range of inhibitors.** (**a**) Capsaicin (Cap) dose response curves for range of AMG-21629 (AMG; potent competitive TRPV1 inhibitor to capsaicin activation) concentrations characterized by SEAP production baseline subtracted by each AMG dose’s respective 0 μM Cap control condition and normalized by peak stimulation observed in the 1.0 μM Cap, 0 nM AMG treatment group (gray dashed line). (**b**) Relative SEAP production with respect to the 0 nM AMG condition (black dashed line) for 0.50 (blue) and 1.0 (red) μM Cap doses as a function of AMG-21629 concentration from (**a**) as highlighted by their corresponding colored boxes. (**c**) Capsaicin dose response curves for range of apocynin (Apo; NADPH oxidase 1 inhibitor) concentrations characterized by SEAP production baseline subtracted by each apocynin dose’s respective 0 μM Cap control condition and normalized by peak stimulation observed in the 0.50 μM Cap, 0 μM Apo treatment group (gray dashed line). (**d**) Relative SEAP production with respect to the 0 μM Apo condition (dashed line) for 0.50 (blue) and 1.0 (red) μM Cap doses as a function of apocynin concentration from (**c**) as highlighted by their corresponding colored boxes. (**e**) Capsaicin dose response curves for range of *N*-acetylcysteine (NAC; ROS scavenger) concentrations characterized by SEAP production baseline subtracted by each NAC dose’s respective 0 μM Cap control condition and normalized by peak stimulation observed in the 1.0 μM Cap, 0 mM NAC treatment group (gray dashed line). (**f**) Relative SEAP production with respect to the 0 mM NAC condition for 0.50 (blue) and 1.0 (red) μM Cap doses as a function of NAC concentration from (**e**) as highlighted by their corresponding colored boxes. All combinatorial dose response conditions were conducted for 4 h at 37°C in a 5.0% CO_2_ incubator with biological replicates *n* = 5. The data for each group in a graph is displayed as either mean ± standard deviation (M ± SD) or mean ± standard error of the mean (M ± SEM) depending on data manipulation. Statistical significance for (**a-f**) was calculated using a two-way ANOVA with Tukey’s multiple comparisons test. Significance level denoted using asterisks (*): * *p* < 0.05, ** *p* < 0.01, *** *p* < 0.001, and **** *p* < 0.0001. For (**b,d,f**), blue and red asterisks correspond to significance level of marked inhibitor concentration for Cap dose of 0.50 and 1.0 μM, respectively, to the 0 M inhibitor gray dashed line in each graph. Solid lines note consecutive inhibitor concentrations that share denoted level of significance to the 0 M inhibitor condition. Blue and red dashed lines correspond to calculated inhibition curves for the 0.50 and 1.0 μM Cap doses, respectively, and are plotted with the points used to calculate them. Inhibition curves and IC_50_ values were calculated using three-parameter inhibitor versus response model constrained between 0 and 1 using the points matched to the plotted curves.

The SEAP production results for these combinatorial studies are shown in Supplementary Fig. 7. For each inhibitor, all of the AMF− conditions were not significantly different from one another (ns), which is similar to the results in Fig. 3d for capsaicin potentiation. This established a consistent baseline for comparing each condition with their own unique controls, rather than the basal response (e.g., Cap−, Inhib−), and were used to generate the relative SEAP production values in Fig. 4. It should also be noted that for all three inhibitors, when AMF stimulation was applied, the Cap−/Inhib−, Cap−/Inhib+, and Cap+/Inhib+ groups were all statistically significant.


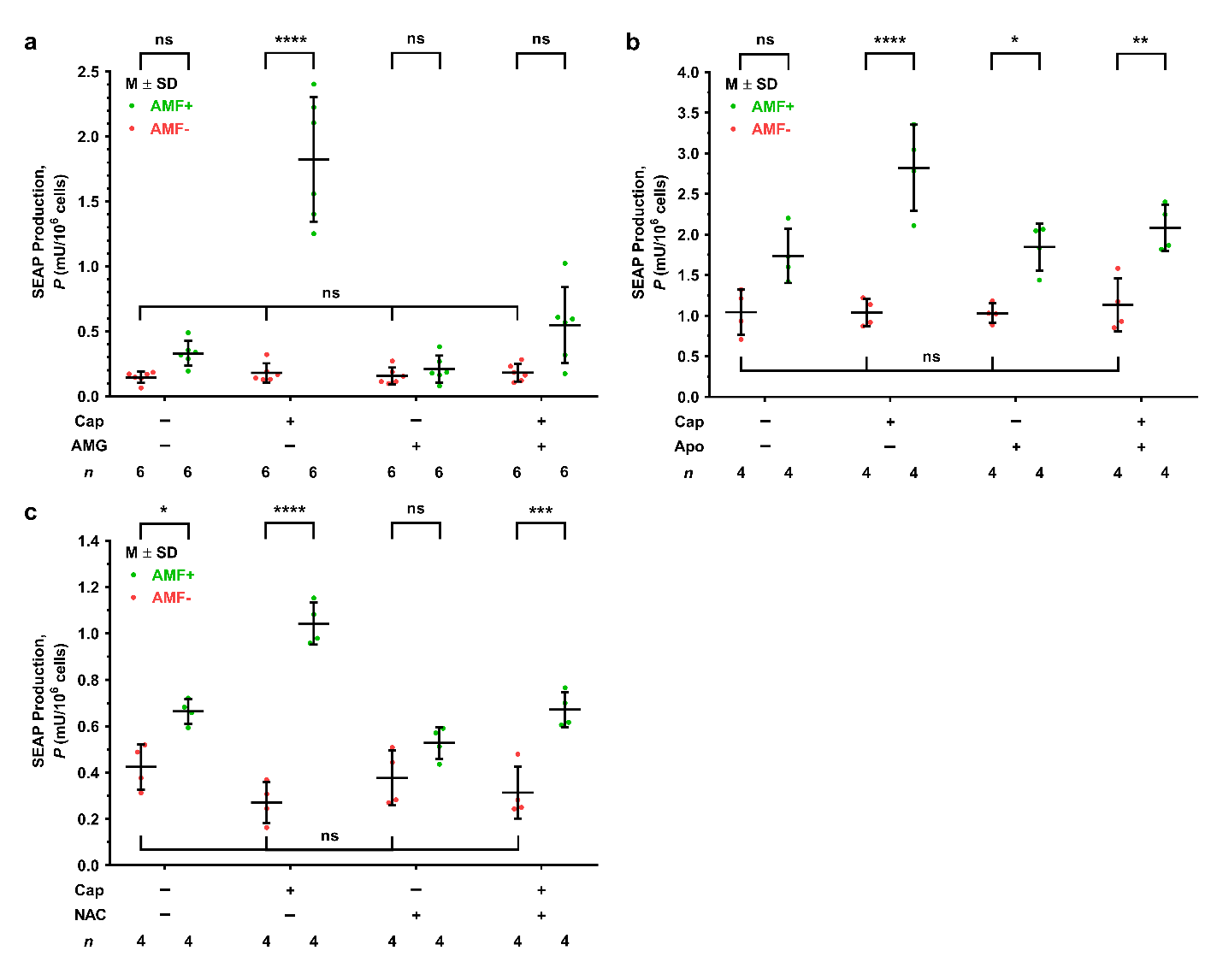


**Supplementary Figure 7.** **Inhibition of magnetogenetic platform response to RF-AMF and RF-AMF/Cap potentiated stimulation.** (**a**) SEAP production under combinatorial RF-AMF (AMF+: ~27.4 mT, 502 kHz AMF), capsaicin (Cap+: 1.0 μM), and AMG-21629 (AMG+: 100 nM) stimulation, potentiation, and inhibition for 2 h. (**b**) SEAP production under combinatorial RF-AMF (AMF+: ~27.4 mT, 502 kHz AMF), capsaicin (Cap+: 1.0 μM), and apocynin (Apo+: 100 μM) stimulation, potentiation, and inhibition for 2 h. (**c**) SEAP production under combinatorial RF-AMF (AMF+: ~27.2 mT, 502 kHz AMF), capsaicin (Cap+: 1.0 μM), and *N*-acetylcysteine (NAC+: 5.0 mM) stimulation, potentiation, and inhibition for 2 h. Sample equilibrium temperature was measured with a thermocouple to set AMF− condition, which was determined to be 32.0°C for all AMF− controls. Values displayed as mean ± standard deviation (M ± SD). Statistical significance for (**a-c**) was calculated using a three-way ANOVA with Tukey’s multiple comparisons test. (**a**) Significant three-way interaction between treatment with AMF, capsaicin, and AMG-21629, *F*(1,40) = 22.43, *p* < 0.0001. (**b**) Significant three-way interaction between treatment with AMF, capsaicin, and apocynin, *F*(1,24) = 4.67, *p* = 0.0409. (**c**) Significant three-way interaction between treatment with AMF, capsaicin, and *N*-acetylcysteine combination, *F*(1,24) = 6.475, *p* = 0.0178. Statistical significance is denoted for the AMF+ treatment of each capsaicin/inhibitor combination to its corresponding AMF− treatment group before normalization. Significance level denoted using asterisks (*): ns *p* ≥ 0.05, * *p* < 0.05, ** *p* < 0.01, *** *p* < 0.001, and **** *p* < 0.0001.

**ROS production in response to capsaicin activation and presence of chemical inhibitors**

To understand the ROS produced as a function of activation and inhibition of the magnetogenetic platform in HEK-nbV1/FtD cells in the absence of AMF, SEAP expression and H_2_O_2_ production were measured in 96-well plate format. As with the preliminary inhibitor studies, these studies were performed in 96-well plates following the aforementioned protocol with the following exceptions. First, at the time of treatment, samples were incubated in 120 μL of Ca-OMEM supplemented with capsaicin-inhibitor combinations to have enough sample to perform assays for both SEAP production and ROS production. ROS production was measured in the form of H_2_O_2_ using the Amplex Red (AR; Sigma-Aldrich) fluorescence assay, which detects AR conversion to the fluorescent molecule resofurin in the presence horseradish peroxidase (HRP; Sigma-Aldrich). Second, after samples were incubated for 4 h with capsaicin-inhibitor combinations, supernatant was collected, centrifuged, and then split into two equal halves with one portion being stored at 4°C until assayed for SEAP production and the other sample being assayed immediately using AR. Briefly, collected supernatant was mixed 50:50 with AR reaction buffer (2 U∙mL^-1^ HRP and 100 μM AR in DPBS). The reaction was allowed to incubate for 30 min at room temperature and shielded from light. ROS production was determined by plate reader fluorescent readings with excitation and emissions wavelengths of 570 nm and 600 nm, respectively, with a 590 nm cutoff filter. Each sample was compared to H­_2_O_2_ standards (Sigma-Aldrich; 30% stock) to determine effective H­_2_O_2_ levels (pmol). For both assays, production was normalized to cell number (mU SEAP per 10^6^ cells and pmol H_2_O_2_ per 10^6^ cells for SEAP and ROS, respectively).


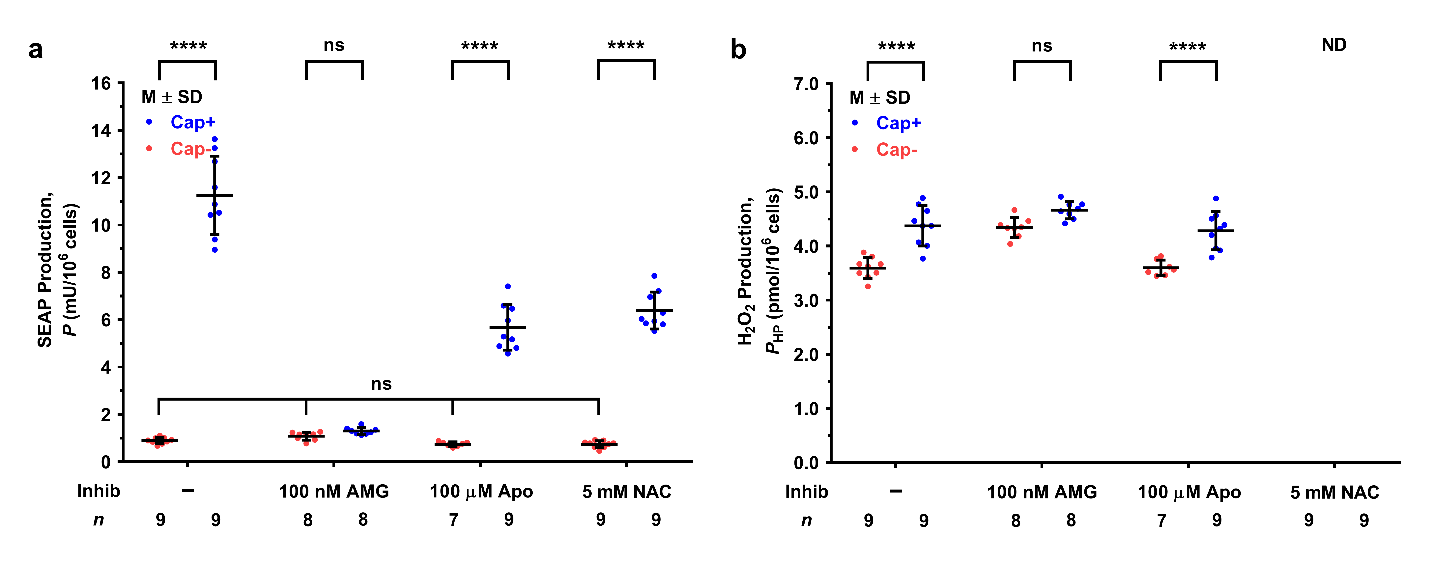


**Supplementary Figure 8.** **Inhibition of magnetogenetic platform response to capsaicin and its effect on ROS generation.** (**a**) SEAP production under combinatorial activation of TRPV1 by capsaicin (Cap+: 0.50 μM) and inhibition by AMG-21629 (AMG+: 100 nM), apocynin (Apo+: 100 μM), and *N-*acetylcysteine (NAC+: 5 mM). (**b**) Corresponding hydrogen peroxide (H_2_O_2_) production to combinatorial stimulation performed in (**a**). Results for NAC were below the limit of detection for the AR assay to measure H_2_O_2_ as it scavenges all types of ROS and is therefore noted as No Data. Values displayed as mean ± standard deviation (M ± SD). NAC+ yielded no data (ND) for the AR assay as NAC scavenges H_2_O_2_. Statistical significance for (**a,b**) was calculated using two-way ANOVA with Tukey’s multiple comparisons test. (**a**) Significant two-way interaction between treatment with capsaicin and presence of an inhibitor on SEAP production, *F*(3,60) = 125.1, *p* < 0.0001. (**b**) Significant two-way interaction between treatment with capsaicin and presence of an inhibitor on H_2_O_2_ production, *F*(2,44) = 3.672, *p* = 0.0335. Significance level denoted using asterisks (*): ns *p* ≥ 0.05, * *p* < 0.05, ** *p* < 0.01, *** *p* < 0.001, and **** *p* < 0.0001.

Comparison of Inhib− and AMG+ reveals that shutting down TRPV1 gating reduces the differential in ROS, as measured by H_2_O_2_, due to capsaicin treatment. This suggests that activation of TRPV1 with capsaicin effects cellular ROS levels as part of the PKC Ca^2+^ signaling cascade. Also consistent with expectations, Inhib− compared to NAC+ shows a reduction in SEAP signaling correlating to reduced Ca^2+^ as a result of sequestering ROS (not measurable due to effect of NAC). Interestingly, Inhib− and Apo+ show similar ROS levels and differentials (Cap− *p* > 0.9999; Cap+ *p* = 0.9739; differentials of 0.78 ± 0.37 and 0.68 ± 0.35 pmol H_2_O_2_ per 10^6^ cells for Inhib− and Apo+, respectively) yet SEAP is significantly reduced. This shows that addition of apocynin does not increase ROS levels, which would be a confounding interaction, during the oxidation step in its intracellular conversion into a NOx assembly inhibitor. This also suggests that inhibiting NOx interferes with PKC Ca^2+^ signaling, but does not reduce overall ROS levels. This is possible whenone considers that other ROS pathways independent of NOx could contribute to measured ROS formation, as well as other ROS species beyond H_2_O_2_ that may play a role and are being lowered.
